# Anti-oncogene PTPN13 inactivation by hepatitis B virus X protein counteracts IGF2BP1 to promote hepatocellular carcinoma progression

**DOI:** 10.1038/s41388-020-01498-3

**Published:** 2020-10-13

**Authors:** Yongcong Yan, Pinbo Huang, Kai Mao, Chuanchao He, Qiaodong Xu, Mengyu Zhang, Haohan Liu, Zhenyu Zhou, Qiming Zhou, Qianlei Zhou, Bing Ou, Qinghua Liu, Jianhong Lin, Ruibin Chen, Jie Wang, Jianlong Zhang, Zhiyu Xiao

**Affiliations:** 1 Department of Hepatobiliary Surgery, Sun Yat-Sen Memorial Hospital, Sun Yat-Sen University, 510120 Guangzhou, China; 2 Guangdong Provincial Key Laboratory of Malignant Tumor Epigenetics and Gene Regulation, Sun Yat-Sen Memorial Hospital, Sun Yat-Sen University, 510120 Guangzhou, China; 3 RNA Biomedical Institute, Sun Yat-Sen Memorial Hospital, Sun Yat-Sen University, 510120 Guangzhou, China; 4 Department of Biliary-Pancreatic Minimally Invasive Surgery, The First Affiliated Hospital of Shantou University Medical College, 515041 Shantou, China; 5 Department of Gastroenterology and Hepatology, The First Affiliated Hospital, Sun Yat-Sen University, 510120 Guangzhou, China; 6 Department of Ultrasound, Sun Yat-Sen Memorial Hospital, Sun Yat-Sen University, 510120 Guangzhou, China

**Keywords:** Liver cancer, Oncogenes, Tumour virus infections, Mechanisms of disease

## Abstract

Hepatitis B x protein (HBx) affects cellular protein expression and participates in the tumorigenesis and progression of hepatitis B virus (HBV)-related hepatocellular carcinoma (HCC). Metabolic reprogramming contributed to the HCC development, but its role in HBV-related HCC remains largely unclear. Tyrosine-protein phosphatase nonreceptor type 13 (PTPN13) is a significant regulator in tumor development, however, its specific role in hepatocarcinogenesis remains to be explored. Here, we found that decreased PTPN13 expression was associated with HBV/HBx. Patients with low PTPN13 expression showed a poor prognosis. Functional assays revealed that PTPN13 inhibited proliferation and tumorigenesis in vitro and in vivo. Further mechanistic studies indicated that HBx inhibited PTPN13 expression by upregulating the expression of DNMT3A and interacting with DNMT3A. Furthermore, we found that DNMT3A bound to the PTPN13 promoter (−343 to −313 bp) in an epigenetically controlled manner associated with elevated DNA methylation and then inhibited PTPN13 transcription. In addition, we identified IGF2BP1 as a novel PTPN13-interacting gene and demonstrated that PTPN13 influences c-Myc expression by directly and competitively binding to IGF2BP1 to decrease the intracellular concentration of functional IGF2BP1. Overexpressing PTPN13 promoted c-Myc mRNA degradation independent of the protein tyrosine phosphatase (PTP) activity of PTPN13. Importantly, we discovered that the PTPN13-IGF2BP1-c-Myc axis was important for cancer cell growth through promoting metabolic reprogramming. We verified the significant negative correlations between PTPN13 expression and c-Myc, PSPH, and SLC7A1 expression in clinical HCC tissue samples. In summary, our findings demonstrate that PTPN13 is a novel regulator of HBV-related hepatocarcinogenesis and may play an important role in HCC. PTPN13 may serve as a prognostic marker and therapeutic target in HBV-related HCC patients.

## Introduction

Liver cancer is the seventh most commonly diagnosed cancer (841,080 new cases annually) and the third leading cause of cancer death (781,631 cancer deaths) worldwide according to the 2018 global cancer statistics [1], and Chinese patients account for nearly half of all patients and deaths [2]. Approximately 75–85% of all primary liver cancers are hepatocellular carcinoma (HCC). Hepatitis B virus (HBV) infection is the major risk factor for HCC, especially in Eastern Asia and Sub-Saharan Africa, outranking hepatitis C virus infection, aflatoxin-contaminated food products, heavy alcohol intake, obesity, smoking, and type 2 diabetes [3]. HBV can cause HCC in the absence of cirrhosis, although most cases of HBV-related HCC (70–90%) occur in patients with cirrhosis [4]. HBV factors, including the hepatitis B x protein (HBx), the pre-S2/S gene and the HBV-spliced protein, have been implicated in liver cancer progression [5]. HBx, a well-known oncogenic protein in HBV-related hepatocarcinogenesis, modulates the expression of important genes and affects the cytoplasmic modulation of signal transduction pathways [6, 7]. Metabolic reprogramming is a selective advantage that supports cancer cell biosynthetic needs, rapid proliferation and tumor growth [8]. However, uncovering the metabolic alterations and mechanisms underlying HBx-mediated tumorigenesis is urgently needed.

Protein tyrosine phosphatases (PTPs) are a class of enzymes that catalyze the dephosphorylation of phosphotyrosyl residues in proteins and include 38 classic PTPs, 21 receptor PTPs, 17 nonreceptor PTPs, and 61 dual-specificity phosphatases [9]. PTPs mainly regulate signal transduction pathways and are involved in important cellular processes [10]. The roles of PTPs, such as PTPRD, PTPRF, PTPRH, PTP1B, PTPN8, PTPN9, PTPN12, and SHP-1/2, in HCC have been widely studied [9]. Recent data have shown that PRL3 transcription induced by GATAD1 activates the AKT signaling pathway and promotes HCC progression [11]. PTPRS inactivation increases the phosphorylation and signaling activity of EGFR to promote HCC metastasis [12]. HBx-enhanced androgen receptor activity is suppressed by sorafenib via the activation of SHP-1 phosphatase, which antagonizes Akt/GSK3β and c-Src pathway activation via HBx [13]. HBx activates the Notch1 pathway, which inhibits dual-specificity phosphatase 1 (DUSP1) and PTEN expression, promoting HCC cell survival [14].

Tyrosine-protein phosphatase nonreceptor type 13 (PTPN13), known as FAP-1 in humans and PTP-BL in mice, negatively regulates FAS-induced apoptosis and NGFR-mediated proapoptotic signaling [15]. PTPN13 promotes pulmonary fibrosis in mice by regulating lung fibroblast resistance to Fas-induced apoptosis [16]. Accumulating evidence suggests that PTPN13 is involved in cancers such as colorectal cancer [17], breast cancer [18], lymphomas [19], and head and neck squamous cell carcinoma [20]. The latest research demonstrates the negative role of PTPN13 in breast tumor invasiveness and highlights its involvement in cell junction stabilization [21]. Given that PTPN13 interacts with putative substrates, such as Ephrin B, STAT4, IRS-1, IκBα, and Her2 [15], PTPN13 may participate in HCC carcinogenesis. PTPN13 expression is downregulated or lost in HCC, and promoter hypermethylation has been detected [22]. In addition, Zhan et al. found that PTPN13 was downregulated in HCC and inhibited epithelial–mesenchymal transition (EMT) through the inactivation of the EGFR/ERK signaling pathway, which suggested that PTPN13 is a tumor suppressor in HCC [23]. We designed this study to elucidate the functions and specific mechanisms underlying the involvement of PTPN13 in HBV-related HCC.

In the present study, we elucidated a novel HCC cell proliferation mechanism involving PTPN13 and insulin-like growth factor 2 mRNA-binding protein 1 (IGF2BP1) that regulates metabolic reprogramming by affecting c-Myc mRNA stability. Low PTPN13 expression was found in HCC cells and tissue samples, especially in HBV-related HCC samples, and was correlated with a poor prognosis in HCC patients. Further studies showed that PTPN13 attenuated cell proliferation and tumorigenesis both in vitro and in vivo. We studied the potential mechanisms by which HBx downregulates PTPN13 expression via DNMT3A-induced DNA methylation. PTPN13-mediated HCC cell growth inhibition by regulating IGF2BP1, thus decreasing the c-Myc mRNA level. Collectively, our results suggested that HBx downregulates PTPN13 expression, which negatively affects cell proliferation and tumorigenesis by interfering with the function of IGF2BP1. These findings may offer novel options for preventing and treating HBV pathogenesis.

## Results

### HBV/HBx downregulates PTPN13 expression

**Fig. 1:**
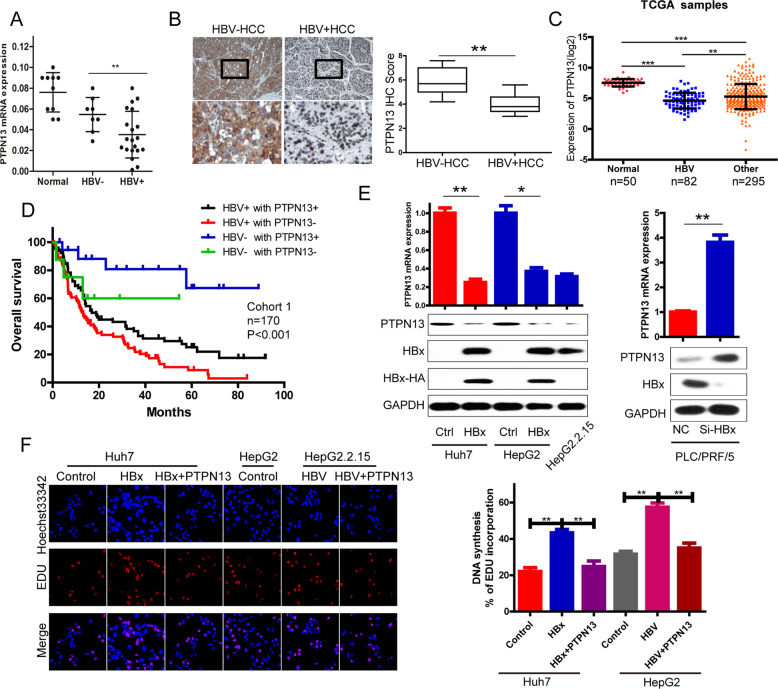
HBx promotes cell proliferation and downregulates PTPN13 expression. **A** qRT-PCR analysis of PTPN13 mRNA levels in 10 normal liver tissue samples, 8 HBV-HCC tissue samples and 20 HBV + HCC tissue samples normalized to GAPDH expression. **B** Left: PTPN13 expression was determined by IHC staining. Representative images of a subset of the tumor specimens are shown. The lower panel shows magnified views (4×), shown in boxes in the upper panel. Right: IHC scores for PTPN13 in the two groups are summarized. **C** The expression levels of PTPN13 were analyzed according to patient HBV positivity. Data are shown after a log2 transformation. **D** Kaplan–Meier survival curve analysis in HCC patients stratified by the combination of the HBV status and PTPN13 expression is shown. **E** The effects of transient HBx overexpression in Huh7 and HepG2 cells, HepG2.2.15 cells (integration of the HBV genome) and HBx knockdown with siRNA in PLC/PRF/5 cells (derived from HBV-infected liver) on the mRNA and protein expression levels of PTPN13. **F** DNA synthesis in stable HBx-overexpressing cells with transient PTPN13 overexpression and stable HBV-overexpressing HepG2.2.15 cells with transient PTPN13 overexpression, as measured by an EdU assay. The data represent the mean ± standard deviation (SD) of three independent experiments (*P < 0.05, **P < 0.01, and ***P < 0.001 compared with the respective control).

PTPN13 expression was significantly lower in HBV-positive HCC tissues (HBV + HCC) than in HBV-negative HCC tissues (HBV−HCC) (Fig. 1A, B and Fig. S1A). We also found low PTPN13 expression in several HCC cell lines with HBV-positive backgrounds, such as HepG2.2.15, PLC/PRF/5, and Hep3B (Fig. S1B). Moreover, 82 HBV-related HCC tissue samples showed lower PTPN13 expression than HCC tissue samples from HBV-negative patients in the Cancer Genome Atlas (TCGA) database (Fig. 1C). Survival analysis revealed that the patients with low PTPN13 expression and an HBV-positive history had the poorest OS among all patients analyzed (Fig. 1D). As mentioned above, low PTPN13 expression was significantly associated with HBV positivity by clinicopathological analysis (Table S1). Next, we found that HBx negatively regulated PTPN13 expression at the RNA and protein levels (Fig. 1E). 5-Ethynyl-2′-deoxyuridine (EdU) assays showed that upregulated PTPN13 expression significantly reversed HBx-induced cell proliferation (Fig. 1F and Fig. S1C). All of these results indicate that PTPN13 expression is strongly downregulated in HBV + HCC patients and HBV-expressing cells.

### HBx inactivates the PTPN13 promoter by interacting with DNMT3A

**Fig. 2:**
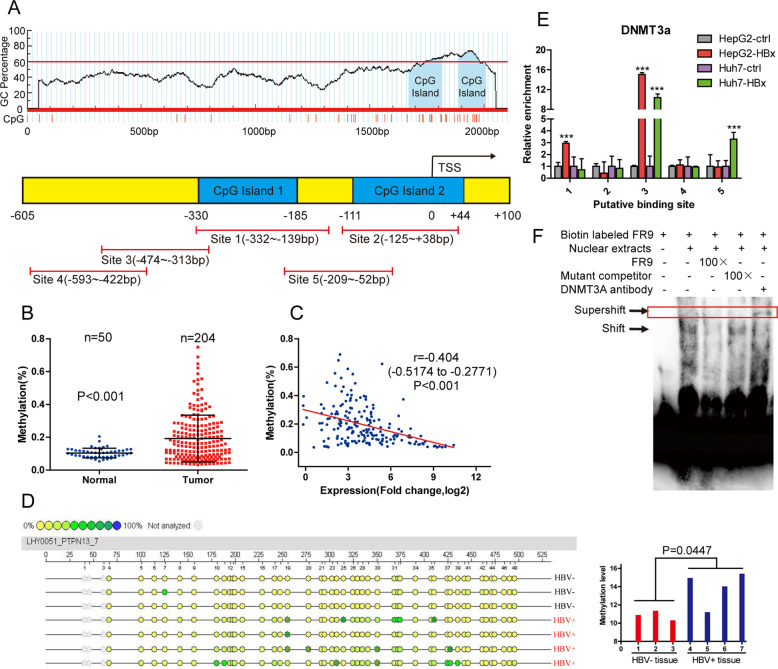
HBx enhances PTPN13 promoter methylation to decrease PTPN13 expression. **A** Upper: CpG sites and CpG islands were identified by promoter sequence analysis. Lower: Five pairs of ChIP-qPCR primers were designed around the two CpG islands. **B** The promoter methylation levels of PTPN13 in the TCGA cohort were analyzed. **C** Correlations between PTPN13 expression and promoter methylation levels in 204 HCC tissue samples in the TCGA cohort were determined and then analyzed by Pearson’s correlation analysis. **D** The PTPN13 promoter methylation levels in HBV-negative HCC tissue samples (*n* = 3) and HBV-positive HCC samples (*n* = 4) were measured by Sequenom MassARRAY quantitative methylation analysis. **E** ChIP assays were performed in HBx stable expression and control cell lines using antibodies against DNMT3A; immunoprecipitated DNA was analyzed by qRT-PCR using primers described in (**A**) for amplifying the DNMT3A-binding regions in the PTPN13 gene promoter. **F** Prepared nuclear extracts were incubated with a biotinylated oligonucleotide probe corresponding to the FR9 region in binding site 3 in the PTPN13 gene promoter to perform an EMSA. Different fold excesses of unlabeled oligonucleotide probes for binding site 3 were used to compete with the interaction between the labeled probe and DNMT3A. The data represent the mean ± SD of three independent experiments (***P < 0.001 compared with the respective control).

To gain further insight into the molecular mechanisms underlying the low PTPN13 expression in HCC, we analyzed PTPN13 DNA sequences and found many CpG sites and two CpG islands in the PTPN13 promoter region (Fig. 2A, upper panel). The PTPN13 promoter was methylated at higher levels in HCC tissue samples than in normal liver tissue samples from TCGA, and the PTPN13 methylation level was inversely correlated with PTPN13 expression (Fig. 2B, C) [24]. Thus, we hypothesized that promoter hypermethylation plays a vital role in PTPN13 expression downregulation in HCC. To verify this hypothesis, we used Sequenom MassARRAY quantitative methylation analysis to determine that the PTPN13 promoter methylation level was higher in 4 HBV + HCC samples than in 3 HBV-HCC samples (Fig. 2D). Next, we investigated whether DNA methyltransferases (DNMTs) participate in the HBV-induced methylation of the PTPN13 promoter. First, we verified that DNMT1 and DNMT3A were highly expressed in a panel of human HCC cell lines, especially those with an HBV-positive background, such as HepG2.2.15, PLC/PRF/5, and Hep3B (Fig. S2A). In addition, western blot analysis revealed that HBx overexpression in HepG2 cells upregulated DNMT1 or DNMT3A expression but downregulated PTPN13 expression in a dose-dependent manner (Fig. S2B, left panel). Moreover, in HepG2 cells stably expressing HBx with low PTPN13 expression, treatment with the DNA methyltransferase inhibitor 5-aza-deoxycytidine (5-aza-dC) reversed PTPN13 expression (Fig. S2B, right panel). We found that only transient DNMT3A overexpression downregulated PTPN13 expression in a dose-dependent manner (Fig. S2C). Exogenous Flag-DNMT3A interacted with HA-HBx in HepG2 cells, demonstrating that HBx physically interacts with DNMT3A (Fig. S2D).

HBx functions as a coactivator that modulates gene transcription without interacting with host DNA sequences. Therefore, we hypothesized that HBx may increase PTPN13 promoter methylation by upregulating DNMT3A expression and interacting with DNMT3A. To further validate this hypothesis, we performed chromatin immunoprecipitation (ChIP; Fig. S3A) using antibodies against DNMT3A in cells stably expressing HBx and control cells, and immunoprecipitated DNA was evaluated by qRT-PCR (Fig. 2A, bottom panel). The results revealed that HBx overexpression increased the interaction between DNMT3A and the PTPN13 promoter in Huh7 and HepG2 cells; however, HBx knockdown weakened this interaction in HepG2.2.15 cells, suggesting that HBx may upregulate DNMT3A expression to suppress PTPN13 expression via methylation (Fig. 2E and Fig. S3B). To find the specific DNMT3A-binding site in the PTPN13 promoter, we divided the 161 bp of DNA pulled down by ChIP into nine overlapping segments and performed an electrophoretic mobility shift assay (EMSA; Fig. S3C). The EMSA indicated that the oligonucleotide probe corresponding to the FR9 region in binding site 3 interacted with PTPN13, and these PTPN13-labeled probe complexes were abrogated by unlabeled oligonucleotides (Fig. 2F and Fig. S3D). Taken together, these results prove that HBx downregulates PTPN13 expression via epigenetic silencing and that DNMT3A physically binds to the PTPN13 promoter at a site −343 to −313 bp upstream of the transcription start site, leading to DNA methylation in hepatoma cells.

### PTPN13 expression is downregulated in HCC patients and hepatoma cells and is inversely correlated with prognosis

**Fig. 3:**
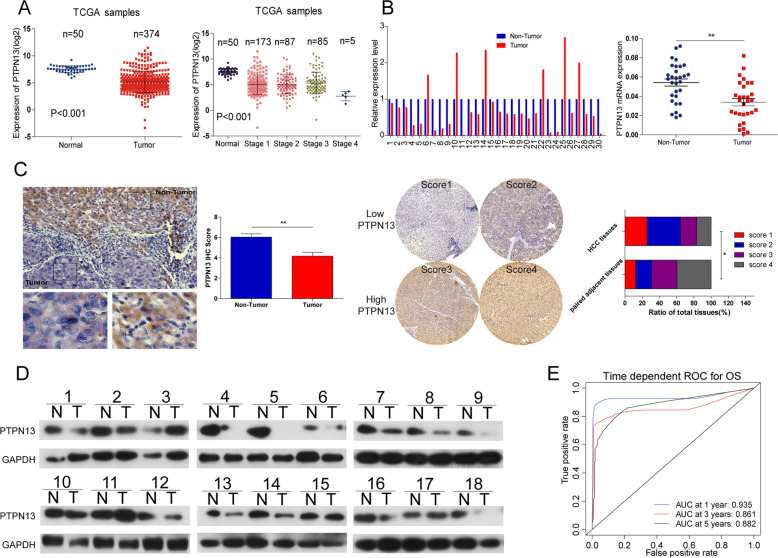
Downregulation of PTPN13 expression is inversely correlated with HCC malignancy. **A** PTPN13 expression levels in 374 HCC tissues and 50 adjacent nontumor liver tissues in the TCGA cohort were analyzed by a *t*-test. Data are shown after a log2 transformation. **B** qRT-PCR was used to analyze PTPN13 mRNA levels in 30 paired samples of human HCC tissues and matched adjacent nontumor liver tissues. **C** Left: Representative images of IHC staining for PTPN13 in HCC tissue and normal liver tissue. Right: Scores indicate the PTPN13 protein levels in representative tumor tissue samples. Scores were calculated by measuring the staining intensity and percentage of stained cells. The PTPN13 protein levels in 170 paired samples were quantified according to the IHC scores. Data are shown as the percentage of total specimens. Values are expressed as the mean ± SD (*P < 0.05, **P < 0.01) for (**A**–**C**). **D** PTPN13 protein levels were significantly lower in paired tumor tissue samples than adjacent noncancerous liver tissue samples based on western blotting. GAPDH served as a loading control (*n* = 18). **E** Time-dependent ROC curves showing the effect of the PTPN13 expression level on HCC patient OS; the area under the curves (AUCs) at 1, 3 and 5 years were calculated.

We found that PTPN13 expression was significantly downregulated in tumor tissue samples compared with normal adjacent tissue samples in the TCGA database and negatively correlated with tumor stage (Fig. 3A). We next analyzed RNA-Seq expression data from TCGA and found that PTPN13 expression changes were not liver-specific, as it was also downregulated in invasive breast carcinoma, thyroid carcinoma, lung adenocarcinoma (LAC), and stomach adenocarcinoma (Fig. S4A). Other studies have reported that PTPN13 expression is markedly lower in LAC than adjacent normal tissue [25, 26] and is an independent prognostic factor of favorable outcome for breast cancer patients [27]. An analysis of three independent cohorts, which included a 179-patient Stanford University cohort (GSE3500), a 45-patient Mount Sinai Liver Cancer Program cohort (GSE6764), and a 445-patient National Cancer Institute cohort (GSE14520), proved that PTPN13 expression was downregulated in HCC tissue **(**Fig. S4B–D). qRT-PCR results revealed that PTPN13 mRNA levels were significantly downregulated in HCC tissues in cohort 1 (Fig. 3B). Consistent with PTPN13 RNA expression, PTPN13 protein expression was high in normal adjacent tissues, as determined by the immunohistochemistry (IHC) analysis of 170 patients **(**Fig. 3C**)** and by western blot analyses of 18 patients (Fig. 3D). Furthermore, multivariate Cox regression analysis demonstrated that high PTPN13 expression was an independent prognostic factor for OS (hazard ratio = 1.742, *p* = 0.003) (Fig. S5). Low PTPN13 expression was significantly associated with HBV positivity, a tumor size ≥5 cm, ≥2 tumors, and venous invasion, as determined by clinicopathological analysis (Table S1). PTPN13 expression was significantly lower at the mRNA and protein levels in the indicated HCC cell lines than the LO2 cell line (Fig. S1B). Time-dependent receiver operator characteristic (ROC) curve analysis suggested that PTPN13 was a stable predictor, and the analysis contained censored survival data (Fig. 3E). Collectively, these results demonstrate that downregulated PTPN13 expression is significantly associated with poor prognoses in HCC patients and strongly suggest that PTPN13 may have a substantial effect on HCC malignancy.

### PTPN13 inhibits cell proliferation and tumorigenesis both in vitro and in vivo

**Fig. 4:**
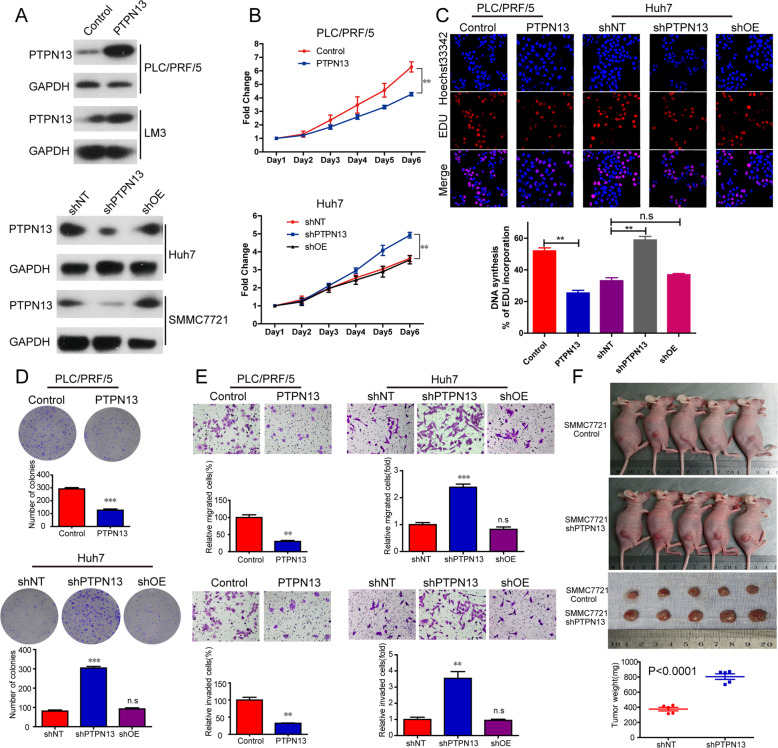
PTPN13 suppresses cell proliferation in vitro and tumor growth in vivo. **A** The confirmation of PTPN13 overexpression (PTPN13), PTPN13 expression knockdown (shPTPN13), and PTPN13 re-expression (shOE) in the indicated HCC cell lines. **B**–**E** The effects of transient PTPN13 overexpression, the control vector, PTPN13 knockdown with shRNA, control shRNA, and PTPN13 re-expression on in vitro proliferation, migration, and invasion, as measured by CCK-8 (**B**), EdU (**C**), colony formation (**D**) and transwell assays (**E**). Data represent the mean ± SD of three independent experiments. **F** The effects of PTPN13 knockdown on tumor growth in nude mice. Tumor images and weights are shown. Data represent the mean ± SD of five samples (**P < 0.01, ***P < 0.001; ns not significant).

We modulated PTPN13 expression to examine its roles in cell proliferation, migration, and invasion in vitro. We overexpressed PTPN13 in PLC/PRF/5 and HCC-LM3 cells with low PTPN13 expression (Fig. 4A). In addition, we constructed a lentivirus carrying shRNA (shPTPN13) to decrease PTPN13 expression. To confirm the knockdown efficiencies of four shRNAs targeting PTPN13 at the protein level in Huh7 and SMMC-7721 cells with high PTPN13 expression, we compared their efficiency with that of a control shRNA (Fig. S6A). shPTPN13 #4, which induced the most significant knockdown, was chosen for subsequent studies. In addition, we reintroduced an engineered PTPN13 cDNA that was not sensitive to shRNA #4 into shPTPN13 (shOE) cells to examine whether PTPN13 re-expression could reverse the changes in HCC cell function induced by shPTPN13 (Fig. 4A). The results of cell counting kit 8 (CCK-8), EdU, colony formation, and transwell assays showed that upregulated PTPN13 expression significantly suppressed the proliferation, migration, and invasion of PLC/PRF/5 and HCC-LM3 cells, while downregulated PTPN13 expression had the opposite effects on Huh7 and SMMC-7721 cells (Fig. 4B–E and Fig. S6B–F). We found that reintroducing PTPN13 increased the proliferation, migration, and invasion of Huh7 and SMMC-7721 cells close to baseline levels (Fig. 4B–E and Fig. S6B–F). To investigate the role of PTPN13 in vivo, SMMC-7721 cells expressing shPTPN13 were subcutaneously injected into nude mice, and tumor volumes and weights were obviously higher in the PTPN13 knockdown group than in the control group (Fig. 4F). Taken together, these results reveal that PTPN13 could be a key factor in antitumor activity, especially inhibiting tumor growth, against HCC in vitro and in vivo.

### PTPN13 physically interacts with IGF2BP1 in HCC cells

**Fig. 5:**
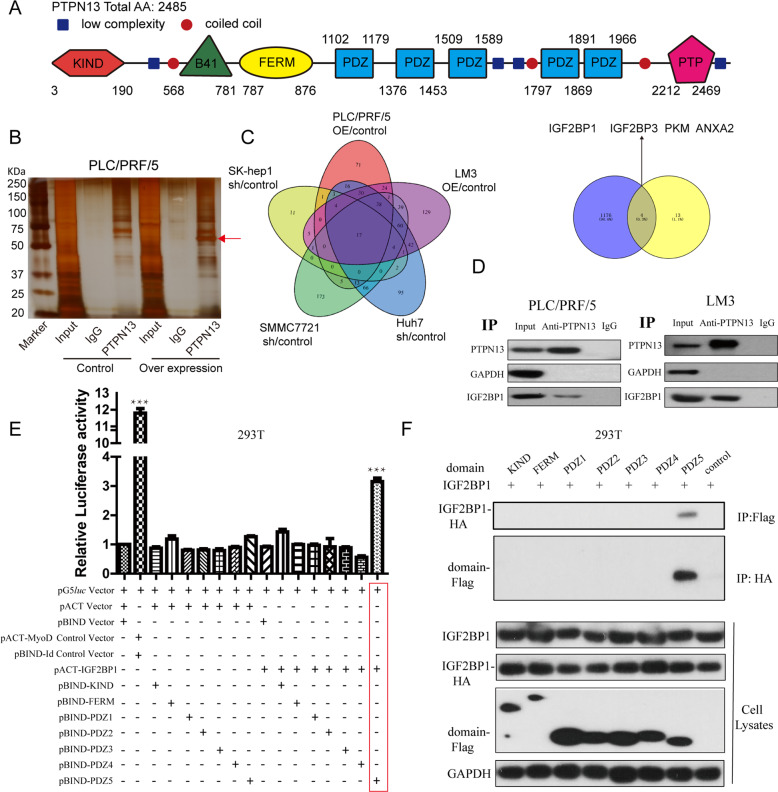
PTPN13 interacts with IGF2BP1 through the fifth PDZ domain. **A** Schematic diagram of the KIND, FERM, PTP, and five PDZ domains in the PTPN13 protein. **B** A CoIP assay was performed using an anti-PTPN13 antibody and IgG control antibody incubated with nuclear extracts of PLC/PRF/5 cells with a transient overexpression vector or a control vector, followed by silver staining. A red arrow indicates IGF2BP1. **C** Left: Interacting proteins based on mass spectrometry after silver staining in the CoIP experiments described in (**B**) in two pairs of transient overexpression cells (PLC/PRF/5-OE vs empty and LM3-OE vs empty) and three pairs of stable knockdown cells (Huh7-sh vs control, SK-hep1-sh vs control, and SMMC-7721-sh vs control). Right: A Venn diagram of protein spectra analysis results and overexpressed mRNAs in the TCGA cohort, which identified four key PTPN13-interacting genes. **D** Immunoblot analysis showed the specific association of IGF2BP1 with PTPN13. Twenty-four hours after transfection, whole-cell lysates were prepared and subjected to immunoprecipitation with an anti-PTPN13 antibody, followed by immunoblotting with an anti-IGF2BP1 antibody. GAPDH was used as a negative control for PTPN13-interacting proteins. **E** Mammalian two-hybrid assay. Plasmids encoding PTPN13 domains (pBIND-domain) and IGF2BP1 (pACT-IGF2BP1) and the pG5luc vector were cotransfected into HEK293T cells. The pBIND-domain plasmids were further used to examine which part of PTPN13 was involved in the physical interaction. pACT-MyoD and pBIND-Id were used as positive controls. Data were normalized to *Renilla reniformis* luciferase activity. **F** CoIP assay and immunoblot analysis of Flag-tagged PTPN13 (domain truncation fragments) immunoprecipitated by HA-tagged IGF2BP1. The data represent the mean ± SD of three independent experiments (***P < 0.001).

According to the amino acid sequence, we examined and visualized the PTPN13 domains, including the catalytic PTP domain at the C terminus, the region with five PDZ domains, the FERM domain, and the KIND domain (Fig. 5A). To further identify the molecular mechanism and binding partners of PTPN13, we performed a coimmunoprecipitation (CoIP) assay with an anti-PTPN13 antibody. An overtly differential band appeared in silver staining and was identified as IGF2BP1 by mass spectrometry (Fig. 5B). Seventeen interacting proteins were identified by protein spectra assays of the indicated paired cell lines (Fig. 5C, left panel; Table S3). Many function-related gene ontology items, such as those associated with biological processes (e.g., poly(A) RNA binding and focal adhesion), were significantly enriched among the 17 interacting proteins (Fig. S7A). We identified 1179 genes with upregulated expression in HCC tissue samples from the TCGA database **(**Fig. S7B) and then identified four proteins, IGF2BP1, IGF2BP3, PKM, and ANXA2, with increased expression in HCC tissue that interacted with PTPN13 (Fig. 5C, right panel). The CoIP of endogenous proteins indicated that PTPN13 interacted with IGF2BP1 (Fig. 5D), IGF2BP3 and PKM but not with ANXA2 (data not shown). Exogenous Flag-IGF2BP1 interacted with PTPN13 in PLC/PRF/5 cells based on immunoprecipitation with an anti-PTPN13, anti-IGF2BP1 or anti-Flag antibody followed by western blotting with a corresponding antibody (Fig. S7C, D). Antibody validation with the shRNA control targeting PTPN13 or IGF2BP1 showed significant peculiarity of antibody through immunofluorescence analysis (Fig. S7E). Immunofluorescence results demonstrated that PTPN13 colocalized with IGF2BP1 in the cytoplasm (Fig. S7F). To investigate the mechanism through which PTPN13 is recruited to IGF2BP1, we performed a mammalian two-hybrid screen of the PTPN13 domains to search for potential IGF2BP1-interacting PTPN13 domains and found that PTPN13 interacted with IGF2BP1 via the fifth PDZ domain (Fig. 5E). The specificity of the interaction was confirmed by CoIP and immunoblot analysis of Flag-tagged PTPN13 (domain truncation fragments) and HA-tagged IGF2BP1 (Fig. 5F). IGF2BP1 possesses 2 RNA-recognition-motif (RRM) domains and 4 hnRNP-Khomology (KH) domains (Fig. S8A). Using CoIP assays with multiple deletions of IGF2BP1, we revealed that deletion of the RRM domains did not affect PDZ5 domain binding, while the KH3–KH4 region of IGF2BP1 was responsible for the interaction with PTPN13 (Fig. S8B). Moreover, we constructed Flag-tagged KH3 and KH4 fragment, then tested KH3 or KH4 individually by CoIP assays with anti-PTPN13 antibody, unfortunately, the results demonstrated that KH3 or KH4 individually can not have an interaction and immunoprecipitation with PTPN13 (Fig. S8C). In addition, we constructed HA-tagged mutant PTPN13 with deletion of PDZ5 (PTPN13ΔPDZ5) fragment, and rescue experiments of PTPN13ΔPDZ5 did not abolish the stimulative effects of PTPN13 silencing on c-Myc expression (Fig. S8D) and cell proliferation (Fig. S8E). Collectively, these data demonstrate that PTPN13 physically interacts with IGF2BP1 in endogenous and exogenous ways, and may regulate cell growth by interacting with IGF2BP1.

### PTPN13 attenuates c-Myc accumulation via IGF2BP1

**Fig. 6:**
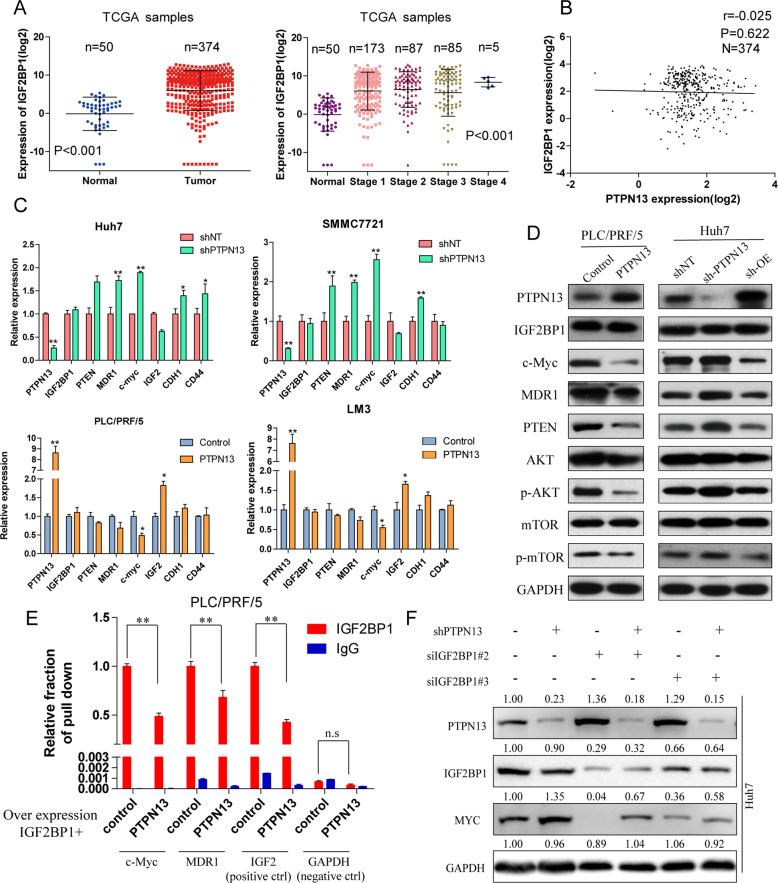
PTPN13 inhibits IGF2BP1 activity to protect target mRNA levels and especially attenuates c-Myc accumulation. **A** The expression levels of IGF2BP1 in 374 HCC tissues and 50 adjacent nontumor liver tissues in the TCGA cohort were analyzed by a *t*-test. Data are shown after a log2 transformation. **B** The correlation between PTPN13 and IGF2BP1 expression in 374 HCC tissue samples. Correlations were then analyzed by Pearson’s correlation analysis. **C** The effects of transient PTPN13 overexpression and a control vector in PLC/PRF/5 and LM3 cells and PTPN13 knockdown with shRNA and a control shRNA in Huh7 and SMMC-7721 cells on the mRNA expression of IGF2BP1 downstream genes are shown. **D** Immunoblot analysis of genes downstream of IGF2BP1 and relevant pathways related to cancer proliferation and metastasis (c-Myc, MDR1, PTEN, p-AKT, and p-mTOR) in PLC/PRF/5 and LM3 cells with transient PTPN13 overexpression and a control vector and Huh7 and SMMC-7721 cells with knockdown and control shRNA. **E** A RIP assay was performed using anti-IGF2BP1 and control IgG antibodies after transient PTPN13 overexpression, followed by qRT-PCR to examine the enrichment of c-Myc, MDR1, IGF2, and GAPDH. IGF2 served as a positive control, while GAPDH served as a negative control. **F** Immunoblot analysis of c-Myc expression in HCC cells expressing PTPN13-specific shRNAs or control cells with or without the transient transfection of IGF2BP1-specific siRNAs. Data represent the mean ± SD of three independent experiments (*P < 0.05, **P < 0.01; ns not significant).

Next, we found that IGF2BP1 expression was significantly higher in tumor tissue samples than normal adjacent tissue samples in TCGA and positively correlated with tumor stage (Fig. 6A) and OS (Fig. S9A). Clinicopathological analysis showed that high IGF2BP1 expression was significantly associated with HBV positivity, a tumor size ≥5 cm, ≥2 tumors, and a high histological grade and tumor-node-metastasis stage in cohort 2 (Table S2). Moreover, IGF2BP1 expression was significantly upregulated in many HCC cell lines (Fig. S9B). To verify IGF2BP1 targets in HCC, we examined selected IGF2BP1 targets by western blotting and found that c-Myc, MDR1, PTEN, and IGF2 were downregulated in response to IGF2BP1 overexpression or expression knockdown (Fig. S9C, D). In addition, there were strong correlations between IGF2BP1 expression and c-Myc or MDR1 in cohort 2 (Fig. S9E). Interestingly, there was no correlation between PTPN13 and IGF2BP1 expression in the TCGA cohort (Fig. 6B) or cohort 2 (Fig. S9E). To elucidate the effects of PTPN13 on IGF2BP1 and its downstream targets, qPCR and immunoblotting assays were performed and showed that PTPN13 did not influence IGF2BP1 expression at the mRNA or protein level but inversely regulated the expression of c-Myc, MDR1, PTEN and other targets (Fig. 6C, D). Considering that IGF2BP1 is a critical RNA-binding protein (RBP), to assess whether PTPN13 influences the mRNA stability of IGF2BP1 downstream targets, we performed a RBP immunoprecipitation (RIP) assay using anti-IGF2BP1 and IgG antibodies after PTPN13 overexpression followed by qRT-PCR and found that PTPN13 overexpression decreased the binding between IGF2BP1 and c-Myc or MDR1 (Fig. 6E). The knockdown of IGF2BP1 expression significantly attenuated c-Myc expression in cells with PTPN13 knockdown, suggesting that PTPN13 participates in the regulation of c-Myc mRNA levels via IGF2BP1 **(**Fig. 6F**)**. Taken together, these results prove that PTPN13 partipcipates in HCC progression by acting as an endogenous competitor of c-Myc mRNA through IGF2BP1, and this signaling was examined in subsequent studies.

### The PTPN13-IGF2BP1-c-Myc axis regulates metabolic reprogramming and thus affects cell proliferation and HCC onset

**Fig. 7:**
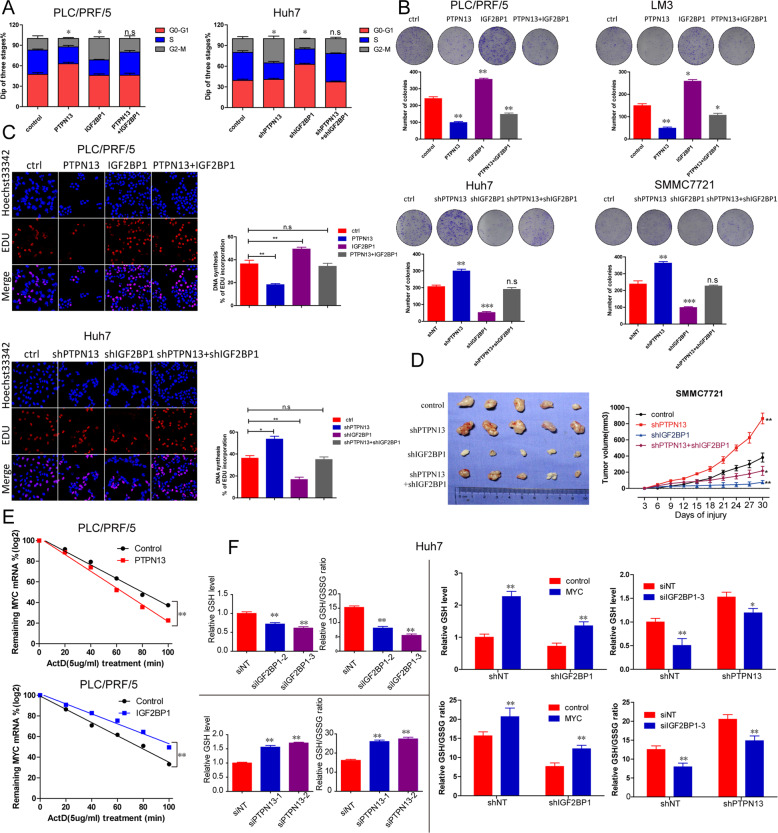
PTPN13 inhibits cell proliferation by inhibiting SSP activation mediated by the IGF2BP1-c-Myc axis. **A** Proliferation profiles of PLC/PRF/5 PTPN13-overexpressing cells transiently transfected with pCDH-IGF2BP1 or the control and PTPN13 interference cells with stable IGF2BP1 knockdown by shRNA or the control were analyzed by cell cycle analysis. **B** Proliferation profiles of the indicated cells described in (**A**) were analyzed by colony formation analysis. **C** Proliferation profiles of the indicated cells described in (**A**) were analyzed by an EdU assay. **D** Proliferative capacity in vivo of PTPN13 interference cells with stable IGF2BP1 knockdown by shRNA or the control were analyzed by subcutaneous tumor formation in nude mice. **E** The remaining c-Myc mRNA expression was measured after treatment with actinomycin D (Act. D) and the knockdown or overexpression of PTPN13 or IGF2BP1 in PLC/PRF/5 cells. **F** The GSH level and GSH/GSSG ratio were determined in Huh7 cells expressing siRNAs targeting IGF2BP1 or PTPN13, stable IGF2BP1 knockdown cells with c-Myc overexpression, and stable PTPN13 knockdown cells with IGF2BP1 knockdown by shRNAs. The data represent the mean ± SD of three independent experiments (*P < 0.05, **P < 0.01, ***P < 0.001; ns not significant).

Given that c-Myc mRNA stability is regulated by PTPN13 via IGF2BP1, we investigated whether PTPN13 suppresses HCC progression in an IGF2BP1-c-Myc-dependent manner. We upregulated IGF2BP1 expression in PLC/PRF/5 PTPN13 cells, revealing that IGF2BP1 overexpression rescued the inhibition of HCC proliferation by PTPN13 overexpression. In addition, we knocked down IGF2BP1 expression in Huh7-shPTPN13 cells, which impaired the ability of shPTPN13 to promote HCC cell proliferation, as determined by EdU and colony formation assays and cell cycle analysis (Fig. 7A–C). IGF2BP1 overexpression significantly affected tumor growth in subcutaneous xenograft models (Fig. S10A). Moreover, based on in vivo results, shIGF2BP1 alone reduced tumor growth, but shPTPN13 did not affect tumor growth after IGF2BP1 silencing compared with control IGF2BP1 expression (Fig. 7D). To block new RNA synthesis, we treated PLC/PRF/5 cells with actinomycin D and then measured c-Myc mRNA levels every 20 min. IGF2BP1 overexpression decreased c-Myc mRNA degradation, while PTPN13 overexpression accelerated c-Myc mRNA degradation (Fig. 7E). Sun et al. [28] revealed that aberrant c-Myc expression leads to enhanced activation of the serine biosynthesis pathway (SSP), an essential part of a metabolic switch, to facilitate HCC progression. Next, we investigated the mechanism by which PTPN13-IGF2BP1-c-Myc might coordinate changes in glutamine metabolism and SSP activation. We found that both the glutathione (GSH) level and the GSH/glutathione disulfide (GSSG) ratio were significantly decreased in Huh7 cells with siRNA-induced IGF2BP1 knockdown (Fig. 7F, top left panel). In contrast, shPTPN13 increased the GSH level and GSH/GSSG ratio (Fig. 7F, bottom left panel). As a result, c-Myc overexpression partially reversed IGF2BP1 expression knockdown-induced decreases in the GSH/GSSG ratio but not the GSH level. In addition, shIGF2BP1 enhanced shPTPN13-induced decreases in the GSH level and GSH/GSSG ratio (Fig. 7F, right panel). Next, we examined what key factors are regulated by the PTPN13-IGF2BP1-c-Myc axis. We found that phosphoserine phosphatase (PSPH) and solute carrier family 7 (SLC7A1) expression was higher in PTPN13 knockdown cells than control cells, which was consistent with the c-Myc expression pattern, in additon, PTPN13 without catalytic activity can inhibit the expression of MYC, PSPH, and SLC7A1 (Fig. S10B). In addition, we found that PSPH and SLC7A1 expression was significantly upregulated in tumor tissue samples compared with normal adjacent tissue samples from TCGA and was positively correlated with OS, Fig. S10C. These results demonstrate that c-Myc plays a role in the PTPN13-mediated regulation of HCC cell proliferation via metabolic reprogramming.

### Downregulated PTPN13 expression is associated with increased c-Myc and metabolism-related enzyme expression in HCC tissues

**Fig. 8:**
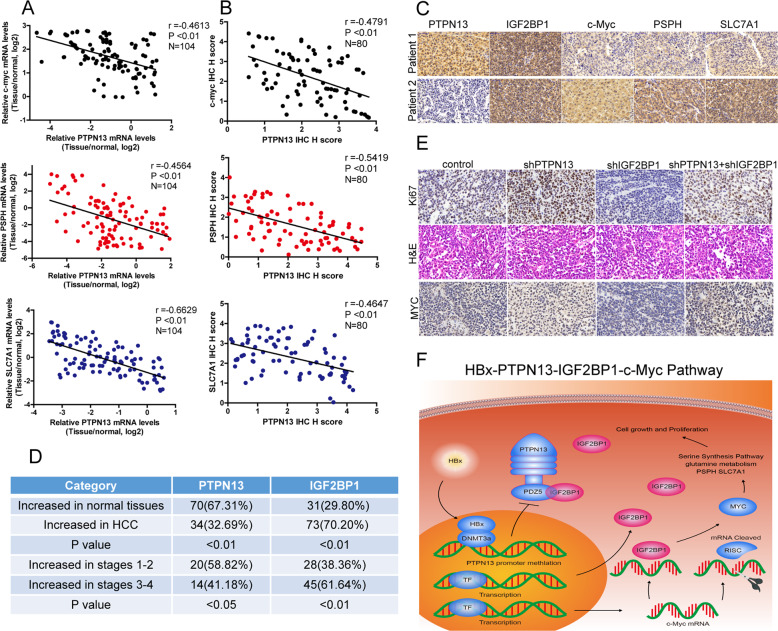
Downregulation of PTPN13 expression is correlated with a positive HBV status, increased c-Myc levels, and elevated glutamine metabolism-related enzyme expression in HCC tissue. **A** Correlations between PTPN13 expression and c-Myc, PSPH, and SLC7A1 expression at the mRNA level in 104 HCC tissue samples were assessed and then analyzed by Pearson’s correlation analysis. **B** Correlations between PTPN13 expression and c-Myc, PSPH, and SLC7A1 expression at the protein level in 80 HCC tissue samples were assessed and then analyzed by Pearson’s correlation analysis. **C** Representative IHC images of PTPN13, IGF2BP1, c-Myc, PSPH, and SLC7A1 expression. **D** PTPN13 and IGF2BP1 protein levels were measured in a tissue microarray by IHC analysis and were analyzed according to tumor stage. **E** Representative IHC images of Ki-67 and c-Myc expression are shown for PTPN13 interference cells with stable IGF2BP1 knockdown by shRNA or control cells in subcutaneous tumors in nude mice. **F** A schematic model for the tumor suppressive function of PTPN13 in HCC is shown. PTPN13 expression was downregulated by high levels of HBx-induced promoter methylation, and PTPN13 decreased the stability of c-Myc mRNA by competitively binding to IGF2BP1, which inhibited c-Myc-associated oncogenic functions involved in HCC cell proliferation, especially the activation of the serine biosynthesis pathway and glutamine metabolism.

To investigate the clinical correlations between PTPN13 and the IGF2BP1 target genes c-Myc, PSPH, and SLC7A1, we performed qPCR analyses of 104 human HCC tissue samples and IHC analyses of 80 HCC tissue samples. We then verified the significant negative correlations between PTPN13 expression and c-Myc, PSPH, and SLC7A1 expression (Fig. 8A–C). Increased PTPN13 expression was observed in only 34 (32.69%) HCC samples compared with 70 (67.31%) normal tissue samples, while increased IGF2PB1 expression was observed in 73 (70.20%) HCC samples compared with only 31 (29.80%) normal tissue samples. For 34 HCC samples with higher PTPN13 expression, there were 20 (58.82%) HCC samples with stage 1–2 disease, but among 73 HCC samples with increased IGF2PB1 expression, there are only 28 (38.36%) HCC samples with stage 1–2 disease (Fig. 8D). Moreover, consistent with the results in HCC cells, PTPN13 knockdown increased c-Myc expression in HCC xenograft models, and IGF2BP1 knockdown reduced c-Myc expression. PTPN13 knockdown together with IGF2BP1 knockdown had no significant effect on c-Myc protein expression (Fig. 8E). Collectively, these results demonstrate that PTPN13 downregulation induced by HBx-induced promoter methylation inhibits HCC proliferation by decreasing c-Myc mRNA stability in an IGF2BP1-dependent manner (Fig. 8F).

### Upregulated IGF2BP1 expression is partially induced by HBx

PTPN13 did not affect IGF2BP1 expression but acted as an endogenous competitor of c-Myc mRNA through IGF2BP1, which resulted in decreased intracellular concentrations of functional IGF2BP1. HBx overexpression not only downregulated PTPN13 expression but also upregulated IGF2BP1 expression in a dose-independent manner (Fig. S11A, B). We found that the increased c-Myc expression induced by HBx was rescued by IGF2BP1 knockdown (Fig. S11C). In addition, in Huh7 cells with the exogenous stable overexpression of HBx or PLC/PRF/5 and Hep3B cells with endogenous HBV overexpression, c-Myc expression depended more on PTPN13 expression than on IGF2BP1 expression, which indicated that the intracellular concentration of functional IGF2BP1 was more significant for c-Myc mRNA stability than the overall IGF2BP1 expression level (Fig. S11D, E). Further clarifying this relationship, stable HBx overexpression in cells with DNMT3A knockdown showed that PTPN13 expression was upregulated and c-Myc expression was downregulated even if IGF2BP1 expression was not significantly altered (Fig. S11F). Taken together, these results show that HBx regulates HCC cell proliferation in an IGF2BP1-dependent manner. HBx upregulates IGF2BP1 expression and increases the intracellular concentration of functional IGF2BP1 by inhibiting PTPN13. Interestingly, PTPN13 is more critical for the HBx-mediated regulation of c-Myc mRNA stability than IGF2BP1.

## Discussion

HCC is one of the most lethal tumors worldwide, and its high mortality and recurrence rates result from uncontrolled cancer cell growth and metastasis [4, 29]. The molecular changes and mechanisms involved in HCC tumorigenesis need to be identified. Here, we revealed that PTPN13 expression was frequently downregulated in HCC and was an independent predictive index for OS. PTPN13 overexpression attenuated cell proliferation and tumorigenesis by regulating metabolic reprogramming. Thus, our results suggest that PTPN13 is a tumor suppressor in HCC and a potential therapeutic target.

PTPN13 is a large protein with a KIND domain, a FERM domain, five PDZ domains and a well-conserved PTP catalytic domain. Previous studies have demonstrated that PTPN13 regulates cellular functions, including survival, proliferation, differentiation, and motility, mainly through protein interactions via its domains [15]. For example, PTPN13 interacts with endogenous TAPP1 or TAPP2 via the first PDZ domain [30]. PTPN13 interacts with TNFRSF6 [31] and TRIP6 [32] via the second PDZ domain and with NGFR and PKN2 [33] via the third PDZ domain. The fourth PDZ domain interacts with PDLIM4, which leads to SRC inactivation via SRC Tyr-419 dephosphorylation [34]. The fourth PDZ domain interacts with ARHGAP29, functioning as a negative regulator of the Rho signal transduction pathway [35]. PTPN13 interacts with SDCCAG3 via the FERM domain to regulate cytokinesis in colon cancers [36]. PTPN13 interacts with FBXL2 to reduce p85β expression, preventing PI3K inhibition by excessive free p85, which can compete with p85-p110 heterodimers [37]. Zhan et al. demonstrated that the tumor suppressor PTPN13 inhibits the malignant behavior of HCC via its phosphatase function and counteracts the activation of EGFR/ERK signaling, as shown by changes in EMT markers and the malignant phenotype induced by EGF [23]. Our study first noted that PTPN13 exhibited anti-HCC activity by interacting with IGF2BP1 via the fifth PDZ domain. PTPN13 bound to IGF2BP1, thus preventing IGF2BP1 from binding the mRNA sequences of its downstream targets. As PTPN13 was a candidate tumor suppressor for HCC, we examined whether the transient re-expression of PTPN13 (shOE) could reverse the enhancement on HCC cell growth induced by silencing PTPN13 expression (shPTPN13). Thus, we reintroduced an engineered PTPN13 cDNA (coding sequence without the 3’untranslated region (3’-UTR)) that was not sensitive to shPTPN13 #4, and found that PTPN13 re-expression was a good way to test its antitumor effect. Finally, we found that reintroducing shOE-PTPN13 returned the increases in cell proliferation close to baseline levels in HCC cells, as other studies have shown [38, 39]. In addition, PTPN13 has been widely suggested to have intrinsic phosphatase activity; however, this interaction was phosphatase-independent. Further studies are needed to evaluate the role of PTPN13 phosphatase activity in HCC progression.

IGF2BP1, also known as IMP-1 or CRD-BP, acts as a posttranscriptional regulator [40]. IGF2BP1 has been reported to have several target RNAs that encode proteins with key roles in tumor transformation and development, such as IGF2, ACTB, CNOT1, GLI1, CD44, MYC, MAPK4, MDR1, KRAS, and β-catenin [40]. Tony et al. suggested that IGF2BP1 functions as a novel potential target to inhibit proliferation and induce apoptosis, partially by downregulating c-MYC mRNA and Ki-67 protein levels during HCC treatment [41]. A novel, liver-specific long noncoding RNA, LINC01093, was recently found to suppress HCC progression by interacting with IGF2BP1 to facilitate GLI1 mRNA degradation [42]. We also found that IGF2BP1 expression was significantly upregulated in HCC tissue and cell lines and positively correlated with tumor stage and OS. RBPs can regulate mRNA fate at the posttranscriptional level via their interactions [43]. IGF2BP1 has two RNA-recognition motifs in the N-terminal region and four KH domains in the C-terminal region [44]. The RNA-recognition motif domain stabilized the protein–RNA complex, and RNA binding is mainly facilitated by the KH domain [42, 45]. Recent reports have indicated that some KH domains are critical for RNA binding [42, 44, 46]. In the present study, we found that the KH3–KH4 region of IGF2BP1 is responsible for the interaction with PTPN13. Unfortunately, the results in Fig. S8C demonstrated that KH3 or KH4 individually can not have an interaction and immunoprecipitation with PTPN13, as for the more detailed physical location that can be combined with PTPN13, we think that more research methods and experiments are needed to excavate and confirm it in the future. To our surprise, qPCR and immunoblotting assays were performed and showed that PTPN13 overexpression did not influence IGF2BP1 expression at the RNA or protein level, but PTPN13 is augmented by the siIGF2BP1#2 (1.36 times) and #3 (1.29 times) in Fig. 6F, it may be a positive feedback regulation between PTPN13 upregulation and IGF2BP1 downregulation. More experiments would be performed focus on this in our further study. Isolated single clones of stable cell lines with PTPN13 and IGF2BP1 double knockdown were selected and expanded, though we had not analyzed clonal variance, but we chose one colony for further analysis and the knockdown efficiency was confirmed by western blotting. In addition, we found that c-Myc was the main downstream target of the PTPN13/IGF2BP1 complex. Interestingly, there was no correlation between PTPN13 and IGF2BP1 expression. Here, we investigated the mechanism by which PTPN13 acts as a protein scaffold to recruit IGF2BP1 to decay c-Myc mRNA. We also found that PTPN13 interacted with IGF2BP3, which requires further investigation.

c-Myc is associated with many oncogenic events, such as tumor cell proliferation, invasion, apoptosis, angiogenesis, drug resistance, and metabolic pathways, which initiate tumorigenesis [47]. Here, we offer a deep understanding of how MYC signaling can drive hepatocarcinogenesis without MYC amplification, a process that probably occurs through IGF2BP1, which is different from the conclusions of other studies on MYC amplification [48]. c-Myc has been extensively studied for its roles in the regulation of glucose, glutamine, and nucleotide metabolism [49, 50], and Gao et al. [28] recently indicated that increased c-Myc expression leads to enhanced SSP activation, an essential part of a metabolic switch, to facilitate HCC progression by transcriptionally upregulating the expression of multiple SSP enzymes. We found that c-Myc overexpression partially attenuated IGF2BP1 knockdown-induced decreases in the GSH/GSSG ratio, which was vital for SSP activation, but unfortunately not in GSH levels. The PTPN13-IGF2BP1-c-Myc axis regulated not only the GSH/GSSG ratio by increasing PSPH expression but also glutamine metabolism via the arginine transporter SLC7A1 to sustain rapid proliferation. However, in this study, we did not find that other SSP enzymes or glutamine transporters, such as PSAT1, PHGDH, SLC3A2 and SLC5A1, were involved. In addition, IGF2BP1 silencing only partially rescued the increased GSH level and GSH/GSSG ratio induced by PTPN13 knockdown, which indicates that PTPN13 has an enormous influence on proliferation and survival and that SSP activation is, at least partially, involved in the PTPN13-mediated regulation of GSH production. However, further study is needed to determine a more detailed mechanism.

The key viral protein HBx is a multifunctional oncoprotein that modulates transcription, signal transduction, cell cycle progression, apoptosis, protein degradation and genetic stability through interactions with host factors [5, 51]. We previously discovered that HBx causes resistance to bortezomib via MEK signaling [5] and regulates the LINC01352-miR-135b-APC axis in tumor progression in HCC [6]. Here, we found that HBx downregulated PTPN13 expression in hepatoma cells. The DNA hypermethylation of CpG islands is a critical epigenetic change that occurs in several malignancies, including HCC [39]. Aberrant PTPN13 hypermethylation has been observed in HCC [22]. Here, PTPN13 expression was downregulated in cell lines that stably expressed HBx, but the expression was significantly restored by treatment with 5-aza-dC. In ChIP and EMSA mechanistic studies, HBx upregulated DNMT3A expression and interacted with DNMT3A. In addition, DNMT3A bound to the PTPN13 promoter (−343 to −313 bp) in an epigenetically controlled manner that was associated with elevated DNA methylation and then inhibited PTPN13 transcription. We further showed that HBx-PTPN13-IGF2BP1-c-Myc-induced metabolic reprogramming was critical for HBx-induced proliferation. PTPN13 did not regulate IGF2BP1 expression but acted as an endogenous competitor of c-Myc mRNA binding to IGF2BP1, which resulted in a decreased intracellular concentration of functional IGF2BP1. HBx overexpression not only downregulated PTPN13 expression but also upregulated IGF2BP1 expression in a dose-independent manner. In this study, we speculated that HBx regulated HCC cell proliferation via multiple mechanisms: HBx upregulated IGF2BP1 mRNA levels, while also enhancing the intracellular concentration of functional IGF2BP1 by inhibiting PTPN13.

In summary, our work elucidated a novel mechanism regulating HCC cell proliferation that involves the antagonism of PTPN13 and IGF2BP1 and the control of c-Myc-induced metabolic reprogramming during hepatocarcinogenesis. The functional characterization of the HBx-DNMT3A-PTPN13-IGF2BP1-c-Myc axis in HBV-induced HCC may be a useful biomarker to guide clinicians during HCC patient management, and molecules in this axis could be promising therapeutic targets to combat HCC.

### Materials and methods

The following detailed methods are listed in Supplementary Materials and methods, including the source of human and mouse tissues, antibodies, cell culture, cell transfection, construction of stable cell lines, qRT-PCR, IHC, Western blot, immunofluorescence, CoIP, ChIP, EMSA, RIP assay, Sequenom massarray quantitative methylation analysis, and data analysis.

## Materials and methods

### Patients and tissue specimens

Two independent HCC cohorts of patients who underwent curative resection between 2009 and 2015 at Sun Yat-Sen Memorial Hospital were enrolled in this study. For cohort 1, 170 pairs of HCC tissue and the corresponding adjacent nontumorous tissues were obtained between January 2009 and December 2012, and 18 paired HCC samples from cohort 1 were randomly selected for western blot analysis. For cohort 2, 104 paired HCC tissue sets collected between January 2013 and December 2015 were used. Follow-up data were obtained and analyzed at the end of October 2018. All samples were confirmed by two pathologists based on the diagnostic criteria proposed by the European Association for the Study of the Liver. No patients received preoperative chemotherapy or radiotherapy. The primary observational index included overall survival (OS), which was defined as the time from diagnosis until death from any cause or the most recent follow-up. The protocol was approved by the ethical committee of Sun Yat-Sen Memorial Hospital, and written informed consent was obtained from all patients before sample collection in accordance with the Declaration of Helsinki. Detailed clinicopathological characteristics of the cohorts are listed in Supplementary Tables S1 and S2.

### Coimmunoprecipitation (CoIP) assay

Cells were lysed in an immunoprecipitation lysis buffer using a commercial kit (Invitrogen, Carlsbad, USA). Total protein (500 μg) was mixed with 2 µg of primary antibody or immunoglobulin G (IgG). Then, the mixture was rotated at 4 °C for 1 h, and protein A/G beads were added, followed by an overnight incubation at 4 °C. The beads were collected and washed three times. A 5× sample loading buffer was added to the beads, which were then boiled for 5 min. The supernatant was analyzed by western blot (Supplementary Table S3).

### QRT-PCR and western blot analysis

Total RNA was isolated from tissues or cultured cell lines using TRIzol reagent (Invitrogen, Carlsbad, USA), and the RNA integrity was verified with the Agilent Bioanalyzer 2100 (Agilent Technologies, Palo Alto, CA). Then, RNA was reverse transcribed into cDNA using the Reverse Transcription Kit (Takara, Dalian, China), and qRT-PCR analyses were performed with Power SYBR Green (Takara, Dalian, China) in triplicate. The data were normalized to GAPDH expression. The qRT-PCR results were analyzed using the 2^−ΔΔCT^ method. All primers are listed in Supplemental Table S4.

Protein was extracted from cells or tissues using RIPA buffer (Beyotime Biotechnology, China), and the concentration was determined using a bicinchoninic acid protein kit (Thermo Scientific, USA). Equal amounts of protein per lane were separated on SDS-PAGE gels and transferred to polyvinylidene fluoride membranes, which were then blocked in 5% nonfat milk in TBST (containing 0.1% Tween-20) for 1 h and incubated with primary antibodies at 4 °C overnight. The membranes were washed with TBST three times, followed by a 1-h incubation with secondary antibodies. Proteins were visualized using an ECL plus western blot detection kit (Pierce Biotechnology). All antibodies are listed in Supplemental Table S5.

### Small interfering RNA knockdown experiments

All protein-coding gene knockdowns were performed in appropriate cells plated in six-well dishes. Experiments were performed with a single transfection of 50 mM experimental siRNA oligos or controls using Lipofectamine 3000 (Invitrogen) in OptiMEM, and the efficiency was determined by qRT-PCR and western blotting. All siRNAs were purchased from GenePharma (Shanghai, China), and their sequences are listed in Supplemental Table S6.

### Chromatin immunoprecipitation (ChIP)-qPCR assay

ChIP assays were performed using the Chromatin Immunoprecipitation Assay Kit (Cell Signaling Technology, Danvers, MA, USA). Cells were crosslinked with 1% formaldehyde, washed with PBS and resuspended in an SDS lysis buffer containing a protease inhibitor cocktail. DNA was sheared into small fragments by sonication and a micrococcal nuclease. The sheared chromatin was immunoprecipitated using the indicated antibodies (anti-DNMT1 and anti-DNMT3A, Abcam, Cambridge, England) overnight at 4 °C. The antibody-chromatin crosslinking was reversed, and the precipitated DNA was purified and detected by qPCR using the primers listed in Supplementary Table S7.

### Electrophoretic mobility shift assay (EMSA)

An EMSA was performed using the LightShift Chemiluminescent EMSA Kit (Pierce, USA) according to the manufacturer’s instructions. Cells were harvested, and nuclear proteins were carefully extracted, followed by determination of the protein content. DNA-binding reactions included biotinylated oligonucleotides and nuclear proteins as well as unlabeled oligonucleotides for competition. Then, the complexes were separated by electrophoresis, transferred onto a positively charged nylon membrane (Millipore, USA) and detected by chemiluminescence. The 161-bp DNA fragment pulled down by ChIP was divided into nine partially overlapping segments. Every segment that could be specifically bound by DNMT3A was screened by the corresponding probe sequence listed in Supplementary Table S8.

## Supplementary information

Supplementary Materials AND Methods

Supplementary Tables

Supplementary Figure legends

Supplementary Figure 1

Supplementary Figure 2

Supplementary Figure 3

Supplementary Figure 4

Supplementary Figure 5

Supplementary Figure 6

Supplementary Figure 7

Supplementary Figure 8

Supplementary Figure 9

Supplementary Figure 10

Supplementary Figure 11
