# Supplementary material for: Anti-oncogene PTPN13 inactivation by hepatitis B virus X protein counteracts IGF2BP1 to promote hepatocellular carcinoma progression
Source: Oncogene. 2020 Oct 13;40(1):28–45. doi: 10.1038/s41388-020-01498-3 (PMC7790756; doi:10.1038/s41388-020-01498-3)
Supplement: Supplementary file 1 — Supplementary Materials AND Methods [file 41388_2020_1498_MOESM1_ESM.docx]

**Mass spectrometry**

Wash with 1ml ultra-pure water twice, 10min for each, followed by digestion and decolorization solution in glue for twice. Add 10mM DTT and incubate for 1h at 56℃, then replaced DTT liquid with 55mM IAM, and incubate at room temperature in darkness for 45min; IAM liquid was discarded, 25mM ammonium bicarbonate was added, and cleaned twice, 10min each time. Discard the above liquid and add decolorizing solution and rinse for 10min, and repeat; then the acetonitrile was vacuum drained. Dilute the enzyme storage solution of 1mg/mL with 25mM ammonium bicarbonate by 15 times and add it into the dehydrated colloidal particles for full absorption. Then add 25mM ammonium bicarbonate to cover the colloidal particles and incubate overnight at 37℃. At last, the digestion was terminated by adding FA with a final concentration of 0.1%.

The supernatant was loaded onto a C18 trap column 5 μL/min for 8min using a LC-20AD nano-HPLC instrument (Shimadzu, Kyoto, Japan) by the autosampler. Then, the peptides were eluted from trap column and separated by an analytical C18 column (inner diameter 75 μm) packed in-house. The gradient was run at 300 nL/min starting from 8 to 35% of buffer B (2% H2O and 0.1% FA in ACN) in 35 minutes, then going up to 60% in 5 minutes, then maintenance at 80% B for 5 minutes, and finally return to 5% in 0.1 min and equilibrated for 10min.

The peptides separated from nanoHPLC were subjected into the tandem mass spectrometry LTQ Orbitrap Velos (Thermo Fisher Scientific, San Jose, CA) for DDA (data-dependent acquisition) detection by nano-electrospray ionization. The electrospray voltage applied was 1.8 kV. Intact peptides were scanned by a range of 350-1500 m/z in the Orbitrap at a resolution of 30,000. Peptides were selected for MS/MS using high-energy collisional dissociation (HCD) operating mode with a normalized collision energy setting of 35. The fragments in MS/MS were scanned at a resolution of 7,500 with the fixed beginning m/z of 100. A data-dependent procedure that alternated between one MS scan followed by 12 MS/MS scans was applied for the 12 most abundant precursor ions above a threshold ion count of 1000 in the MS survey scan with a following Dynamic Exclusion duration of 15 s. Automatic gain control (AGC) was used to optimize the spectra for full MS target at 1e6 and MS2 target at 5e4.

The protein identification uses experimental MS/MS data and aligns them with theoretical MS/MS data from database to obtain results.

**Cell lines**

The human HCC cell lines HepG2.2.15 (integration of the HBV genome), and Huh7 and the normal human hepatic cell line LO2 were purchased from the cell bank of the Committee on Type Culture Collection (CTCC) of the Chinese Academy of Sciences (Shanghai, China). HepG2, Sk-Hep-1, MHCC-97L, LM3, MHCC-97H and L02 cells were purchased from Zhong Qiao Xin Zhou Biotechnology Co., Ltd. (Shanghai, China). SMMC-7721 cells were purchased from Genechem Co., Ltd. (Shanghai, China). PLC/PRF/5 and Hep3B (derived from HBV-infected liver tissue) cells were purchased from the American Type Culture Collection (ATCC; Manassas, VA, USA). Most cell lines were cultured in Dulbecco’s modified Eagle’s medium (DMEM; Gibco, Carlsbad, CA, USA) supplemented with 10% fetal bovine serum (FBS; Gibco, Carlsbad, CA, USA), but SMMC-7721 cells were cultured in RPMI 1640 medium (Gibco, Carlsbad, CA, USA) supplemented with 10% FBS. All cells were maintained in a humidified incubator at 37°C in a 5% CO_2_ atmosphere.

**Immunohistochemistry (IHC) and immunofluorescence (IF)**

Fixed HCC and paired adjacent normal liver tissue samples and xenograft tumor specimens from nude mice were sectioned at 5-µm thickness. The tissue samples were deparaffinized, subjected to antigen retrieval with sodium citrate and incubated with 3% hydrogen peroxide for 15 minutes at room temperature. Then, they were blocked with goat serum for 15 minutes at 37°C and incubated with the indicated antibody overnight at 4°C. Subsequently, the relevant secondary antibody (Santa Cruz Biotechnology, Inc., Santa Cruz, CA, US) was added, and the sections were incubated for 1 hour at room temperature. The slides were incubated with a streptavidin-HRP conjugate and then counterstained with hematoxylin. The sections were scanned, and the images were then digitalized and analyzed using Image-Pro Plus 5.1 software.

Adherent cells were washed twice with cold phosphate-buffered saline (PBS) and fixed with 4% phenylmethylsulfonyl fluoride (PMSF) for 15 minutes at room temperature. Then, the cells were treated with 0.3% Triton X-100 for cell permeabilization at room temperature for 15 minutes, incubated with a blocking buffer (3% BSA in PBS) for 1 hour, and incubated with a primary antibody at 4°C overnight and with a secondary antibody at room temperature for 1 hour. Cell nuclei were stained with DAPI. Images were captured using a laser confocal microscope (LSM 800, Zeiss, Germany).

**Determination of the glutathione (GSH) level and reduced and oxidized GSH (GSH/GSSG) ratio**

Intracellular GSH levels and the GSH/GSSG ratio were measured using assay kits (V6611 and V6911) purchased from Promega.

**Plasmids and stable cell line generation**

Plasmid vectors for transfection were extracted using a DNA Midiprep kit (Qiagen, Hilden, Germany). A retroviral packaging system was purchased from Clontech. Viral supernatants were collected 72 hours after 293T cells were transfected with the plasmid vector. Lentiviral particles were concentrated using a LentiX™ Concentrator (Clontech, Mountain View, CA, USA) at 4°C overnight. The retroviral vector pMSCV-eGFP containing HBx-HA and the pcDNA3.1-HBx-HA plasmid were generated as described previously described [1, 2]. PcDNA3.1-DNMTs-3xFlag plasmids were obtained by inserting DNMT1-3xFlag or DNMT3a-3xFlag into the pcDNA3.1(+) vector (Invitrogen). The whole open-reading frame (ORF) of PTPN13 was engineered into pcDNA3.1(+)(GUANGZHOU IGE BIOTECHNOLOGY LTD). PDZ5 was encodes by a nucleotide sequence of 228bp, HA tagged mutant PTPN13 with deletion of PDZ5 (PTPN13ΔPDZ5) fragment was constructed and engineered into pcDNA3.1(+). All constructs were confirmed by Sanger DNA sequencing. shRNA targeting sequences against PTPN13 were cloned into the GV112 hU6-MCS-CMV-Neomycin vector, while shIGF2BP1 was cloned into the GV248 hU6-MCS-CMV-Puromycin-EGFP vector (GeneChem). shRNA targeting sequences are listed in Table S6.

Constructions of stable cell lines:

For shPTPN13+transient PTPN13 overexpression, shPTPN13 #4 targeted the 3’-UTR region of PTPN13, which means that it did not affect the exogenous overexpression of PTPN13. ShRNA lentivirus was made by cotransfecting the shRNA #4 vector with pLP/VSVG, pLP1/Gag-pol and pLP2/Rev in a 2:1:1:1 ratio into 293T cells and collecting and concentrating the supernatant. The target cells were infected with filtered lentivirus plus 10 ug/mL polybrene (Sigma) for 24 hours and treated with 4 µg/ml neomycin (InvivoGen) for 10 days. Isolated single colonies were selected and expanded. Then, HCC cells with stable PTPN13 knockdown were transfected with the PTPN13 plasmid and its empty vector control using Fugene HD (E2311, Roche) according to the manufacturer’s protocol.

For shPTPN13+shIGF2BP1, shPTPN13 stable knockdown cell lines were transfected with the shIGF2BP1 construct and treated with 2 µg/mL puromycin and 4 µg/ml neomycin (InvivoGen) at the same time. After continuous antibiotic marker selection for more than 10 days, isolated single colonies were selected and expanded. The infection efficiency was confirmed by western blotting. All the cell lines were used within 20 passages, and fresh aliquots were thawed every 2 months.

**Cell proliferation, colony formation and transwell assays**

Cell proliferation rates were measured using cell counting kit-8 (CCK-8) (Dojindo) and EdU assays. Cells were seeded at 1x10^3^ cells per well in 96-well plates. Cell proliferation was assessed at the appropriate time points (24, 48, 72, 96 and 120 hours); 10 µl of CCK-8 solution was added to each well, followed by incubation for 4 hours at 37°C. The absorbance was measured at 450 nm. Each six-replicate experiment was repeated three times. The EdU Fluor 594 Imaging Kit (Ribo Biology, Inc., Guangzhou, China) was used for the EdU-based cell proliferation assay according to the manufacturer’s instructions. Adherent cells were collected and washed with PBS. The cells were fixed with cold 95% ethanol overnight at 4°C, stained with 50 µg/ml propidium iodide (BD Pharmingen TM, Heidelberg, Germany) for 30 minutes, and detected by flow cytometry.

For the colony formation assay, each well of a 6-well culture plate was seeded with 500 cells, and three wells were used for each treatment group. After incubation at 37°C for 10 days, the cells were fixed with methanol and stained with a Giemsa solution. Visible colonies were then manually counted under a microscope.

Cell motility and invasion were measured with transwell and Matrigel chamber plates, respectively (24-well format; 8-µm pore size; Corning Costar, New York, USA). A total of 5x10^5^ cells were seeded on the membranes in serum-free medium; 600 µl of medium supplemented with 10% FBS was added to the corresponding lower chamber. After incubation for 24 or 48 hours, the cells on the upper surface of the filters were removed with cotton swabs, and the cells on the lower surface were fixed in 4% paraformaldehyde and stained with 1% crystal violet. Then, the cells were imaged, and five random fields were evaluated.

**Xenograft tumor growth**

For an in vivo tumorigenesis study, nude mice (athymic BALB/c-nu/nu, female, 4 to 6 weeks old) were housed in specific pathogen-free (SPF) environments and were randomly grouped. The animal care and experimental protocols were performed in accordance with a protocol approved by the Animal Research Committee of Sun Yat-Sen University. A total of 3x10^6^ cells were suspended and mixed with Matrigel (BD Biosciences, Bedford, MA, USA) at a 1:3 volume ratio in 100 µL of serum-free DMEM and injected subcutaneously into mice. Tumor volumes were measured every 3 days using a caliper and calculated using the standard formula V = shortest diameter^2^×longest diameter/2. The mice were euthanized when animals exhibited either 30 days after subcutaneous injection or large tumors (volume > 1500 mm3) or obvious signs of discomfort. The tumor was removed, photographed and weighed before being fixed in 10% buffered formalin and analyzed by hematoxylin and eosin (H&E) staining and IHC.

**Mammalian two-hybrid assay**

A mammalian two-hybrid system was used to further confirm the interaction between the domains of PTPN13 and IGF2BP1. HEK293T cells were transiently cotransfected with pG5luc, the pACT-IGF2BP1-HA vector or pACT-HA and a pBIND-PTPN13 domain-3xFLAG plasmid carrying KIND (7-570), FERM (2359-2628), PDZ1 (3304-3537), PDZ2 (4126-4359), PDZ3 (4525-4767), PDZ4 (5389-5607), PDZ5 (5671-5898), or PTP (6634-7407) for 48 hours. Then, the cells were lysed and subjected to luciferase activity assays using a Dual-Glo system (Promega, WI). When pACT-IGF2BP1-HA was used, the luciferase value was labeled L1, and the Renilla value was labeled R1. When pACT-HA was used, the luciferase value was labeled L2, and the Renilla value was labeled R2. The relative luciferase activation was calculated as follows: (L1/R1) / (L2/R2).

**RNA-binding protein (RBP) immunoprecipitation (RIP) assay**

RIP assays were performed using a Millipore EZ-Magna RIP RNA-Binding Protein Immunoprecipitation kit (Millipore, #17-701). Cells were harvested with RIP lysis buffer. The IGF2BP1 protein in the supernatant, protein G beads, and an anti-IGF2BP1 or IgG antibody were mixed to perform the immunoprecipitation. RNAs that bound to IGF2BP1 were eluted and quantified by qRT-PCR, while total RNA was used as an input control.

**The cancer genome atlas (TCGA) and gene expression omnibus (GEO) database mining**

We downloaded level 3 data, which contained the high-throughput sequencing data of mRNAs from 374 HCC samples and 50 normal samples, from the TCGA (https://portal.gdc.cancer.gov/). Clinical data, such as patient prognosis and basic clinical information, are available from the Data Coordinating Center. For PTPN13 promoter methylation analysis, data were available from the MethHC database (<http://methhc.mbc.nctu.edu.tw/>). Kaplan-Meier survival analysis of patients in the TCGA cohort stratified by IGF2BP1 expression was obtained by gene expression profiling interactive analysis (GEPIA; http://gepia.cancer-pku.cn/index.html). Microarray data and patient clinical information were downloaded from the GEO database ([https://www.ncbi.nlm.nih.gov/geo/).](https://www.ncbi.nlm.nih.gov/geo/).%20In%20addition,%20we)

**Sequenom massarray quantitative methylation analysis**

Sequenom MassARRAY quantitative methylation analysis was performed using the MassARRAY Compact System ([www.sequenom.com](http://www.sequenom.com)). DNA (5 μg) extracted from tissue was subjected to sodium bisulfite treatment, which converts every unmethylated cytosine into a uracil, while every methylated cytosine remains a cytosine. Then, 1 μl of bisulfite-treated DNA was amplified, desalted and spotted on a 384-pad SpectroCHIP, followed by spectral acquisition with a MassARRAY Analyzer Compact MALDI-TOF MS (Sequenom). The average methylation level was calculated.

**Statistical analysis**

Statistical analysis was performed using IBM SPSS Statistics version 24 (SPSS Inc., Chicago, IL, USA), R version 3.3.4 ([www.R-project.org](http://www.R-project.org)) and GraphPad Prism version 5.0 (GraphPad Software, San Diego, CA). Data are expressed as the mean ± standard deviation (x ±SD). The χ2 test was used to compare categorical variables, while Student’s t test or one-way analysis of variance was used to analyse the parametric variables (two-tailed). Kaplan-Meier survival curves were used to estimate OS in different groups, and survival differences were assessed by a two-sided log-rank test. Pearson’s correlation analysis was performed to determine the correlation between two variables. All experiments were carried out with at least three replicates. All statistical tests were two-sided, and a P value <0.05 was considered statistically significant.

**References:**

1. Huang P, Zhuang B, Zhang H et al. Hepatitis B Virus X Protein (HBx) Is Responsible for Resistance to Targeted Therapies in Hepatocellular Carcinoma: Ex Vivo Culture Evidence. Clin Cancer Res 2015; 21: 4420-4430.

2. Huang P, Xu Q, Yan Y et al. HBx/ERalpha complex-mediated LINC01352 downregulation promotes HBV-related hepatocellular carcinoma via the miR-135b-APC axis. Oncogene 2020.
