# Supplementary material for: Anti-oncogene PTPN13 inactivation by hepatitis B virus X protein counteracts IGF2BP1 to promote hepatocellular carcinoma progression
Source: Oncogene. 2020 Oct 13;40(1):28–45. doi: 10.1038/s41388-020-01498-3 (PMC7790756; doi:10.1038/s41388-020-01498-3)
Supplement: Supplementary file 2 — Supplementary Tables [file 41388_2020_1498_MOESM2_ESM.docx]

**Table S1. Comparison of clinicopathological characteristics between Negative or Positive PTPN13 expression in HCC patients from SYMH.**

| Clinical Characteristics | | PTPN13 expression level | |  |  | P value |  |
| --- | --- | --- | --- | --- | --- | --- | --- |
|  |  | Negative | Positive | OR | 95%CI |  |  |
| Gender | Man/Woman | 72/13 | 69/16 | 0.779 | 0.349-1.738 | 0.541 | |
| Age(years) | Mean±SD | 48.81±12.13 | 51.96±11.69 | - | - | 0.086 | |
| Hepatitis history | HBV+  HBV- | 77  8 | 66  19 | 0.361 | 0.148-0.878 | **0.021** | |
| AFP  (ng/ml) | ﹤100  ≥100 | 29  56 | 36  49 | 0.705 | 0.379-1.312 | 0.269 | |
| Cirrhosis | Positive  Negative | 58  27 | 62  23 | 0.634 | 0.338-1.187 | 0.153 | |
| Tumor size(cm) | <5  ≥5 | 25  60 | 38  47 | 0.515 | 0.274-0.970 | **0.039** | |
| Tumor number | <2  ≥2 | 51  34 | 63  22 | 0.524 | 0.273-1.004 | **0.050** | |
| Venous invasion | Positive  Negative | 48  37 | 32  53 | 0.465 | 0.252-0.860 | **0.014** | |
| Tumor  Grade | I/II  III/IV | 64  21 | 67  18 | 0.819 | 0.400-1.677 | 0.584 | |
| pTNM | I/II  III/IV | 48  37 | 45  40 | 1.153 | 0.630-2.110 | 0.644 | |

Significance was determined by Chi-square test. OR, odds ratio; 95% CI, 95% confidence interval.p <0.05 was considered statistically significant.

**Table S2. Comparison of clinicopathological characteristics between Negative or Positive IGF2BP1 expression in HCC patients from SYMH.**

| Clinical Characteristics | | IGF2BP1 expression level | |  |  | P value |
| --- | --- | --- | --- | --- | --- | --- |
|  |  | Negative | Positive | OR | 95%CI |  |
| Gender | Man/Woman | 42/10 | 47/5 | 2.238 | 0.708-7.078 | 0.163 |
| Age(years) | Mean±SD | 51.19±12.63 | 46.77±10.99 | - | - | 0.060 |
| Hepatitis history | HBV+  HBV- | 35  17 | 47  5 | 4.566 | 1.537-13.565 | **0.004** |
| AFP  (ng/ml) | ﹤100  ≥100 | 21  31 | 14  38 | 1.839 | 0.805-4.200 | 0.146 |
| Cirrhosis | Positive  Negative | 36  16 | 35  17 | 0.915 | 0.401-2.090 | 0.833 |
| Tumor size(cm) | <5  ≥5 | 27  25 | 14  38 | 2.931 | 1.292-6.652 | **0.009** |
| Tumor number | <2  ≥2 | 41  11 | 25  27 | 4.025 | 1.704-9.509 | **0.001** |
| Venous invasion | Positive  Negative | 27  25 | 23  29 | 1.362 | 0.629-2.946 | 0.432 |
| Tumor  Grade | I/II  III/IV | 37  15 | 26  26 | 2.467 | 1.098-5.543 | **0.027** |
| pTNM | I/II  III/IV | 34  18 | 24  28 | 2.204 | 1.000-4.840 | **0.048** |

Significance was determined by Chi-square test. OR, odds ratio; 95% CI, 95% confidence interval.p <0.05 was considered statistically significant.

**Table S3. Detailed information about 17 proteins interacting with PTPN13.**

| Hits | Protein ID | Description | Protein Mass(Da) |
| --- | --- | --- | --- |
| 1 | sp\|Q9NZI8\|IF2B1_HUMAN | Insulin-like growth factor 2 mRNA-binding protein 1 | 63441.17493 |
| 2 | sp\|O00425\|IF2B3_HUMAN | Insulin-like growth factor 2 mRNA-binding protein 3 | 63665.56072 |
| 3 | sp\|Q13283\|G3BP1_HUMAN | Ras GTPase-activating protein-binding protein 1 | 52132.07556 |
| 4 | sp\|P61978\|HNRPK_HUMAN | Heterogeneous nuclear ribonucleoprotein K | 50944.4019 |
| 5 | sp\|P60709\|ACTB_HUMAN | Actin, cytoplasmic 1 | 41709.72951 |
| 6 | sp\|P38646\|GRP75_HUMAN | Stress-70 protein, mitochondrial | 73634.77131 |
| 7 | sp\|P35908\|K22E_HUMAN | Keratin, type II cytoskeletal 2 epidermal | 65393.21601 |
| 8 | sp\|P35527\|K1C9_HUMAN | Keratin, type I cytoskeletal 9 | 62026.81367 |
| 9 | sp\|P14618\|KPYM_HUMAN | Pyruvate kinase PKM | 57900.02229 |
| 10 | sp\|P13645\|K1C10_HUMAN | Keratin, type I cytoskeletal 10 | 58791.69264 |
| 11 | sp\|P11021\|GRP78_HUMAN | 78 kDa glucose-regulated protein | 72288.43496 |
| 12 | sp\|P07437\|TBB5_HUMAN | Tubulin beta chain | 49638.97066 |
| 13 | sp\|P07355\|ANXA2_HUMAN | Annexin A2 | 38579.81417 |
| 14 | sp\|P07237\|PDIA1_HUMAN | Protein disulfide-isomerase | 57080.67209 |
| 15 | sp\|P04406\|G3P_HUMAN | Glyceraldehyde-3-phosphate dehydrogenase | 36030.39723 |
| 16 | sp\|P04264\|K2C1_HUMAN | Keratin, type II cytoskeletal 1 | 65998.99984 |
| 17 | sp\|O43707\|ACTN4_HUMAN | Alpha-actinin-4 | 104788.4721 |

**Table S4. Sequences of primer sets used in quantitative qRT-PCR.**

| Gene | RefSeq Summary | Primers for qRT-PCR | Sequence(5'−3') |
| --- | --- | --- | --- |
| PTPN13 | NM_080685 | Sense | ttggaatgacactgtattgggg |
|  |  | Antisense | ccaagcagtatgctgttgagat |
| IGF2BP1 | NM_006546 | Sense | TAGTACCAAGAGACCAGACCC |
|  |  | Antisense | GATTTCTGCCCGTTGTTGTC |
| PTEN | NM_000314 | Sense | TGCAGAAAGACTTGAAGGCG |
|  |  | Antisense | AGTTCTAGCTGTGGTGGGTT |
| MDR1 | NM_000927 | Sense | CCAAAGTCAACAAGGAGTGC |
|  |  | Antisense | TCTTCAACAGTGGTTTATCGCA |
| HBx | NC_003977.2 | Sense | ACCTCTCTTTACGCGGTCTC |
|  |  | Antisense | CCAACTCCTCCCAGTCCTTA |
| c-Myc | NM_002467 | Sense | AGCGACTCTGAGGAGGAAC |
|  |  | Antisense | CGTAGTTGTGCTGATGTGTG |
| IGF2 | NM_001127598 | Sense | CGATGCTGGTGCTTCTCACC |
|  |  | Antisense | GTCACAGCTGCGGAAACAGC |
| CDH1 | NM_004360 | Sense | GCCGAGAGCTACACGTTCAC |
|  |  | Antisense | GTCGAGGGAAAAATAGGCTG |
| CD44 | NM_000610 | Sense | CGCCAAACACCCAAAGAAGA |
|  |  | Antisense | TTCCTGCTTGATGACCTCGT |
| DNMT1 | NM_001130823 | Sense | cctagccccaggattacaagg |
|  |  | Antisense | actcatccgatttggctctttc |
| DNMT3a | NM_022552 | Sense | CCTGGTGATTGGAGGCAGTC |
|  |  | Antisense | CATGCAGGAGGCGGTAGAAC |
| GAPDH | NM_002046 | Sense | GAAGGTGAAGGTCGGAGTCA |
|  |  | Antisense | TGACAAGCTTCCCGTTCTCA |

**Table S5. Antibodies used in this paper.**

| Antibodies | Source | Identifier |
| --- | --- | --- |
| PTPN13 | Santa Cruz | sc-15356 |
| IGF2BP1 | Cell Signaling Technology | #8482 |
| DNMT1 | Abcam | ab13537 |
| DNMT3a | Abcam | ab2850 |
| HBx | Abcam | ab39716 |
| c-Myc | ImmunoWay | YT0991 |
| PTEN | Santa Cruz | sc-7974 |
| MDR1 | Abcam | ab168337 |
| AKT | Cell Signaling Technology | #2920 |
| p-AKT | Cell Signaling Technology | #4060 |
| mTOR | Cell Signaling Technology | #2983 |
| p-mTOR | Cell Signaling Technology | #5536 |
| GAPDH | Santa Cruz | sc-25778 |
| PSPH | Proteintech | 14513-1-AP |
| SLC7A1 | Abcam | ab60303 |
| HA | Abcam | ab9110 |
| Flag | Abcam | ab49763 |
| Alexa Fluor 555 | Abcam | ab150074 |
| Alexa Fluor 647 | Abcam | ab150115 |

**Table S6. siRNA/shRNA sequences used in this paper.**

| **Gene** | | **Sequence(5'−3')** |
| --- | --- | --- |
| **siRNA** | |  |
| HBx-1 | Sense | GAGGCUGUAGGCAUAAAUU |
| HBx-2 | Sense | GCACUUCGCUUCACCUCUG |
| IGF2BP1-1 | Sense | CCGGGAGCAGACCAGGCAA |
| IGF2BP1-2 | Sense | UGAAUGGCCACCAGUUGGA |
| IGF2BP1-3 | Sense | CCGGGAGCAGACCAGGCAA |
| PTPN13-1 | Sense | GCAGUAACAGUGCGGACUU |
| PTPN13-2 | Sense | CCAGGAGACCGUUUGAUAU |
| PTPN13-3 | Sense | GCCUUCUACUCCUGUGCAU |
| DNMT1-1 | Sense | GGAGCUGUUCUUGGUGGAU |
| DNMT1-2 | Sense | GGAAAUACUCCGACUACAU |
| DNMT1-3 | Sense | GGAACUUUGUCUCCUUCAA |
| DNMT3a-1 | Sense | CCUCAGAGCUAUUACCCAA |
| DNMT3a-2 | Sense | GAUUAUUGAUGAGCGCACA |
| DNMT3a-3 | Sense | CAGUCCACUAUACUGACGU |
| **shRNA** | |  |
| PTPN13-1 | Sense | CCGGGCCACGGTCTATTCTTACTAACTCGAGTTAGTAAGAATAGACCGTGGCTTTTTG |
|  | Antisense | AATTCAAAAAGCCACGGTCTATTCTTACTAACTCGAGTTAGTAAGAATAGACCGTGGC |
| PTPN13-2 | Sense | CCGGCCTTTGGATCAGTGTCTAATTCTCGAGAATTAGACACTGATCCAAAGGTTTTTG |
|  | Antisense | AATTCAAAAACCTTTGGATCAGTGTCTAATTCTCGAGAATTAGACACTGATCCAAAGG |
| PTPN13-3 | Sense | CCGGCAGATCTTAGGGATGATTAAACTCGAGTTTAATCATCCCTAAGATCTGTTTTTG |
|  | Antisense | AATTCAAAAACAGATCTTAGGGATGATTAAACTCGAGTTTAATCATCCCTAAGATCTG |
| PTPN13-4 | Sense | CCGGGCTACTTTAACCTATGATAATCTCGAGATTATCATAGGTTAAAGTAGCTTTTTG |
|  | Antisense | AATTCAAAAAGCTACTTTAACCTATGATAATCTCGAGATTATCATAGGTTAAAGTAGC |
| IGF2BP1-1 | Sense | CCGGTGAAGATCCTGGCCCATAATACTCGAGTATTATGGGCCAGGATCTTCATTTTTG |
|  | Antisense | AATTCAAAAATGAAGATCCTGGCCCATAATACTCGAGTATTATGGGCCAGGATCTTCA |
| IGF2BP1-2 | Sense | CCGGCTCCAAAGTTCGTATGGTTATCTCGAGATAACCATACGAACTTTGGAGTTTTTG |
|  | Antisense | AATTCAAAAACTCCAAAGTTCGTATGGTTATCTCGAGATAACCATACGAACTTTGGAG |
| IGF2BP1-3 | Sense | CCGGACGCTTAGAGATTGAACATTCCTCGAGGAATGTTCAATCTCTAAGCGTTTTTTG |
|  | Antisense | AATTCAAAAAACGCTTAGAGATTGAACATTCCTCGAGGAATGTTCAATCTCTAAGCGT |
| IGF2BP1-4 | Sense | CCGGGCAGTGGTGAATGTCACCTATCTCGAGATAGGTGACATTCACCACTGCTTTTTG |
|  | Antisense | AATTCAAAAAGCAGTGGTGAATGTCACCTATCTCGAGATAGGTGACATTCACCACTGC |

**Table S7. Sequences of primer in PTPN13 promoter sets used in Chromatin immunoprecipitation assay.**

| Sites | Location in promoter region | | Sequence(5'−3') |
| --- | --- | --- | --- |
| 1 | -332~-139bp | Sense | GCTGCATTTTCAACTTGGCC |
|  |  | Antisense | AGCTCCACCTCCTCCCTAG |
| 2 | -125~+38bp | Sense | CCAATGAGGTCGAGGGGAG |
|  |  | Antisense | gtgtcaccctCGcctcag |
| 3 | -474~-313bp | Sense | AAGCGCCCCGTGAGAATATA |
|  |  | Antisense | GCCAAGTTGAAAATGCAGCC |
| 4 | -593~-422bp | Sense | GGAAAACTCACCGGGCTTTT |
|  |  | Antisense | CCTTACTCTTCGCTGCCTCT |
| 5 | -209~-52bp | Sense | GCACTGGTTGTCATGGCAA |
|  |  | Antisense | CTGTCACTCAACCATTCCCC |

**Table S8. Probe sequences sets used in EMSA.**

| Probe Name | Probe sequence |
| --- | --- |
| B-F1 | 5´-aagcgccccgtgagaatatagaaacaaggctg-3´-biotin |
| B-R1 | 5´-cagccttgtttctatattctcacggggcgctt-3´-biotin |
| B-F2 | 5´-tatagaaacaaggctgagaggcagcgaagagt-3´-biotin |
| B-R2 | 5´-actcttcgctgcctctcagccttgtttctata-3´-biotin |
| B-F3 | 5´-agaggcagcgaagagtaagggaggagccagca-3´-biotin |
| B-R3 | 5´-tgctggctcctcccttactcttcgctgcctct-3´-biotin |
| B-F4 | 5´-aagggaggagccagcacttacgcatttttcac-3´-biotin |
| B-R4 | 5´-gtgaaaaatgcgtaagtgctggctcctccctt-3´-biotin |
| B-F5 | 5´-cttacgcatttttcactctcctcctcactccc-3´-biotin |
| B-R5 | 5´-gggagtgaggaggagagtgaaaaatgcgtaag-3´-biotin |
| B-F6 | 5´-tctcctcctcactccctagggtcatagttttg-3´-biotin |
| B-R6 | 5´-caaaactatgaccctagggagtgaggaggaga-3´-biotin |
| B-F7 | 5´-tagggtcatagttttgattctgaagttagaag-3´-biotin |
| B-R7 | 5´-cttctaacttcagaatcaaaactatgacccta-3´-biotin |
| B-F8 | 5´-attctgaagttagaagtgtgggggtcgtcggc-3´-biotin |
| B-R8 | 5´-gccgacgacccccacacttctaacttcagaat-3´-biotin |
| B-F9 | 5´-tgtgggggtcgtcggctgcattttcaacttggc-3´-biotin |
| B-R9 | 5´-gccaagttgaaaatgcagccgacgacccccaca-3´-biotin |
| F9 | 5´-tgtgggggtcgtcggctgcattttcaacttggc-3´ |
| R9 | 5´-gccaagttgaaaatgcagccgacgacccccaca-3´ |
| M-F | 5´-agtcagtcagtcagtcagtcagtcagtcagtc-3´ |
| M-R | 5´-gactgactgactgactgactgactgactgact-3´ |
