# Supplementary material for: Anti-oncogene PTPN13 inactivation by hepatitis B virus X protein counteracts IGF2BP1 to promote hepatocellular carcinoma progression
Source: Oncogene. 2020 Oct 13;40(1):28–45. doi: 10.1038/s41388-020-01498-3 (PMC7790756; doi:10.1038/s41388-020-01498-3)
Supplement: Supplementary file 3 — Supplementary Figure legends [file 41388_2020_1498_MOESM3_ESM.docx]

**Supplementary Figure legends**

**Figure S1.HBx influences PTPN13 expression.Related to Figure 1**

(A) Normal rabbit IgG primary reagent control was used at the same IgG concentration as anti-PTPN13 for the IHC analysis of paraffin sections containing HCC and adjacent normal tissues.

(B) qRT-PCR analysis of PTPN13 mRNA levels and immunoblot analysis of PTPN13 protein levels in HCC cell lines.

(C) Immunoblot analysis to verify the overexpression levels of HBx and PTPN13 in Figure 1F in the indicated cell lines. The data represent the mean ± SD of three independent experiments (*P < 0.05, **P < 0.01).

**Figure S2. DNMT1 and DNMT3A expression upregulation induced by HBx inhibits PTPN13 expression. Related to Figure 2**

(A) qRT-PCR analysis of mRNA levels and immunoblot analysis of protein levels of DNMT1 and DNMT3A in HCC cell lines.

(B) Left: The relative expression levels of PTPN13, DNMT1 and DNMT3A, as measured by western blotting, in HepG2 cells after the transient transfection of the HBx vector at two doses. Right: Induction of PTPN13 gene expression in HepG2 cells with stable HBx overexpression by 5-aza-dC treatment.

(C) Immunoblot analysis of PTPN13 protein levels after DNMT1 or DNMT3A overexpression by transient transfection.

(D) CoIP assay and immunoblot analysis of Flag-tagged DNMT3A immunoprecipitated by HA-tagged HBx. Whole-cell lysates were prepared and subjected to immunoprecipitation with anti-Flag and anti-HA antibodies, followed by immunoblotting with anti-HBx/HA and anti-DNMT3A/Flag antibodies. The data represent the mean ± SD of three independent experiments (**P < 0.01, ***p < 0.001 compared with the respective control).

**Figure S3. HBx downregulates PTPN13 expression via promoter methylation at the -343~-313 bp site. Related to Figure 2**

(A) The optimal ratio of micrococcal nuclease concentration to the number of cells for the digestion of cross-linked chromatin DNA into 150- to 900-bp fragments for ChIP assays was explored.

(B) ChIP assays were performed in HBx knockdown cell lines with siRNA and an siRNA control by using antibodies against DNMT3A. Immunoprecipitated DNA was measured by qRT-PCR using primers described in Figure 2A for amplifying the DNMT3A-binding regions in the PTPN13 gene promoter.

(C) The 161-bp DNA fragment (-474~-313 bp) pulled down by ChIP was divided into 9 segments that overlapped to design EMSA probes.

(D) Prepared nuclear extracts were incubated with a biotinylated oligonucleotide probe for the third putative binding site (-474~-313 bp; described in D) to perform an EMSA. The data represent the mean ± SD of three independent experiments (**P < 0.01 compared with the respective control).

**Figure S4. PTPN13 expression analyses in various cancers in TCGA cohorts and three Gene Expression Omnibus (GEO) cohorts. Related to Figure 3**

(A) The expression of PTPN13 in the TCGA cohort was analyzed in various cancer types, as indicated. Data are shown as medians with extreme values. The Mann-Whitney U test was used for statistical analysis.

(B-D) The expression of PTPN13 was analyzed in three GEO datasets, which included GSE3500, the Chen-HCC cohort (GSE3500, GSE6764, and GSE14520), Wurmbach-HCC cohort (GSE6764) and Roessler-HCC cohort (GSE14520). Data were obtained from the ONCOMINE database ([www.oncomine.org](http://www.oncomine.org)). Data are shown as medians with extreme values. The Mann-Whitney U test was used for statistical analysis.

**Figure S5. Univariate and multivariate Cox regression analyses and forest plots of the HRs and 95% confidence intervals of OS for HCC patients in cohort 1. Related to Figure 3**

**Figure S6. PTPN13 suppresses cell proliferation and metastasis in vitro. Related to Figure 4**

(A) Confirmation of the knockdown efficiency of four PTPN13-specific shRNAs in Huh7 and SMMC7721 cells. shPTPN13 #4 had the highest knockdown efficiency for PTPN13. shPTPN13 #4 targeted the 3’-UTR region of PTPN13, which means that it did not affect the exogenous overexpression of PTPN13, and was then used to construct PTPN13 re-expression cell lines (shOE, also see Fig. 4A).

(B-F) The effects of transient PTPN13 overexpression, the control vector, PTPN13 expression knockdown with shRNA, the control shRNA and re-expression on in vitro proliferation, migration and invasion, as measured by EdU (B), CCK-8 (C), colony formation (D), transwell migration (E) and migration (F) assays. The data represent the mean ± SD of three independent experiments (*P < 0.05, **P < 0.01, ***P < 0.001; ns, not significant).

**Figure S7. PTPN13 interacts with IGF2BP1. Related to Figure 5**

(A) GO biological function analysis of 17 potential PTPN13-interacting proteins using the Database for Annotation, Visualization, and Integrated Discovery (DAVID).

(B) Hierarchical clustering plots of differentially expressed mRNAs (DEGs). The lower horizontal axis represents samples, and the upper horizontal axis represents clusters of samples. The left vertical axis represents clusters of DEGs, and the right vertical axis shows DEG names. Red represents upregulated DEGs, and green represents downregulated DEGs.

(C) CoIP assay was performed using an anti-PTPN13 antibody and IgG control antibody incubated with the nuclear extracts of PLC/PRF/5 cells that transiently overexpressed exogenous PTPN13 and IGF2BP1, followed by silver staining. The red arrow indicates IGF2BP1, and the black arrow indicates PTPN13.

(D) CoIP assay and immunoblot analysis. PLC/PRF/5 cells transiently overexpressing exogenous PTPN13 and IGF2BP1 lysates were prepared and subjected to immunoprecipitation with anti-PTPN13, anti-IGF2BP1, anti-Flag and anti-IgG antibodies, followed by immunoblotting with anti-IGF2BP1 and anti-PTPN13 antibodies.

(E) Antibody validation with the shRNA control targeting PTPN13 or IGF2BP1 was evaluated by immunofluorescence analysis.

(F) Colocalization between PTPN13 and IGF2BP1 was evaluated by immunofluorescence analysis. Normal rabbit IgG and mouse IgG served as negative controls for anti-PTPN13 (rabbit Ab) and anti-IGF2BP1 (mouse Ab).

**Figure S8. PTPN13 interacts with KH3-KH4 region of IGF2BP1 via the fifth PDZ domain. Related to Figure 5**

(A) Schematic diagram of the RRM and KH domains on the IGF2BP1 protein

(B) Immunoblot analysis of Flag-tagged IGF2BP1 (full-length or domain truncation fragments) after the CoIP assay using an anti-PTPN13 antibody.

(C) Immunoblot analysis of Flag-tagged KH3, KH4 and 408-577 fragments, then tested KH3 or KH4 individually, and KH3-KH4 fragment by CoIP assays with anti-PTPN13 antibody.

(D) Immunoblot analysis of c-Myc expression in HA tagged mutant PTPN13 with deletion of PDZ5 (PTPN13ΔPDZ5) fragment or wild type PTPN13 in PLC/PRF/5 cells.

(E) The effects of PTPN13ΔPDZ5, wild type PTPN13 and the control vector on in vitro proliferation by colony formation assays. The data represent the mean ± SD of three independent experiments (**P < 0.01; ns, not significant).

**Figure S9. IGF2BP1 expression is upregulated in HCC and enhances the expression of several oncogenes. Related to Figure 6**

(A) Kaplan-Meier curve analysis for the OS of the high- and low-level groups stratified by IGF2BP1 expression in the TCGA cohort. The P value of the log rank test (two-sided, P = 0.026) is shown.

(B) Immunoblot of IGF2BP1 protein in the indicated HCC cell lines.

(C) IGF2BP1 overexpression vector and control vector were transduced into Huh7 and SMMC7721 cells, and western blotting was performed to confirm the overexpression of IGF2BP1 and changes in its downstream genes.

(D) IGF2BP1 siRNA and control siRNA were transduced into Hep3B and PLC/PRF/5 cells, and western blotting was performed to confirm the knockdown efficiency of three IGF2BP1-specific siRNAs and their effects on downstream gene expression.

(E) Correlations between PTPN13 and IGF2BP1 expression and between IGF2BP1 and c-Myc or MDR1 expression in 104 HCC tissue samples analyzed by qPCR. The correlations were then analyzed by Pearson’s correlation analysis.Data represent the mean ± SD of three independent experiments (*P < 0.05; ns, not significant).

**Figure S10. Upregulation of PSPH or SLC7A1 expression correlates with HCC prognosis. Related to Figure 7**

(A) The effects of IGF2BP1 overexpression on tumor growth in nude mice. Tumor pictures and tumor volume are shown. Data represent the mean ± SD of four samples for the control group and five samples for the IGF2BP1 group.

(B) Immunoblotting evaluated the effects of PTPN13 knockdown and mutating PTPN13 (mut-PTPN13) by deleting the PTP fragment (PTPN13ΔPTP) on the expression of c-Myc and related genes in Huh7 and SMMC7721 cells.

(C) Upper: The expression levels of PSPH or SLC7A1 were analyzed in the TCGA cohort. The data were obtained from TANRIC ([http://ibl.mdanderson.org/tanric/design/basic/query.html](http://ibl.mdanderson.org/tanric/_design/basic/query.html)). Data are shown as medians with interquartile ranges. The Mann-Whitney U test was used for statistical analysis. Lower: Kaplan-Meier curve analysis was used to evaluate OS in patients stratified according to high and low expression of PSPH or SLC7A1. The P value of the log rank test is shown.

**Figure S11. IGF2BP1 upregulation is partly induced by HBx.**

(A) The effects of transient HBx overexpression in Huh7 cells and endogenous HBx in HepG2.2.15 cells on the expression of PTPN13, IGF2BP1 and other related genes by qRT-PCR analysis.

(B) The effects of transient and stable HBx overexpression in Huh7 and HepG2 cells on the expression of PTPN13 and IGF2BP1 by immunoblot analysis.

(C) Immunoblot analysis of c-Myc expression in stable HBx-overexpressing cells with IGF2BP1 knockdown with siRNA and the corresponding control in Huh7 and HepG2 cells.

(D) Immunoblot analysis of IGF2BP1 and c-Myc expression in stable HBx-overexpressing Huh7 cells with transient PTPN13 overexpression and an empty control.

(E) Immunoblot analysis of IGF2BP1 and c-Myc expression in HBx endogenous overexpression with PTPN13 transient overexpression and empty control in PLC/PRF/5 and Hep3B (HBV-positive, derived from HBV-infected liver) cells.

(F) Immunoblot analysis of IGF2BP1 and c-Myc expression in stable HBx-overexpressing cells with DNMT3A knockdown by siRNA in Huh7 and HepG2 cells. Data represent the mean ± SD of three independent experiments (*P < 0.05, **P < 0.01; ns, not significant).
