# Supplementary figures and images for: Anti-oncogene PTPN13 inactivation by hepatitis B virus X protein counteracts IGF2BP1 to promote hepatocellular carcinoma progression
Source: Oncogene. 2020 Oct 13;40(1):28–45. doi: 10.1038/s41388-020-01498-3 (PMC7790756; doi:10.1038/s41388-020-01498-3)

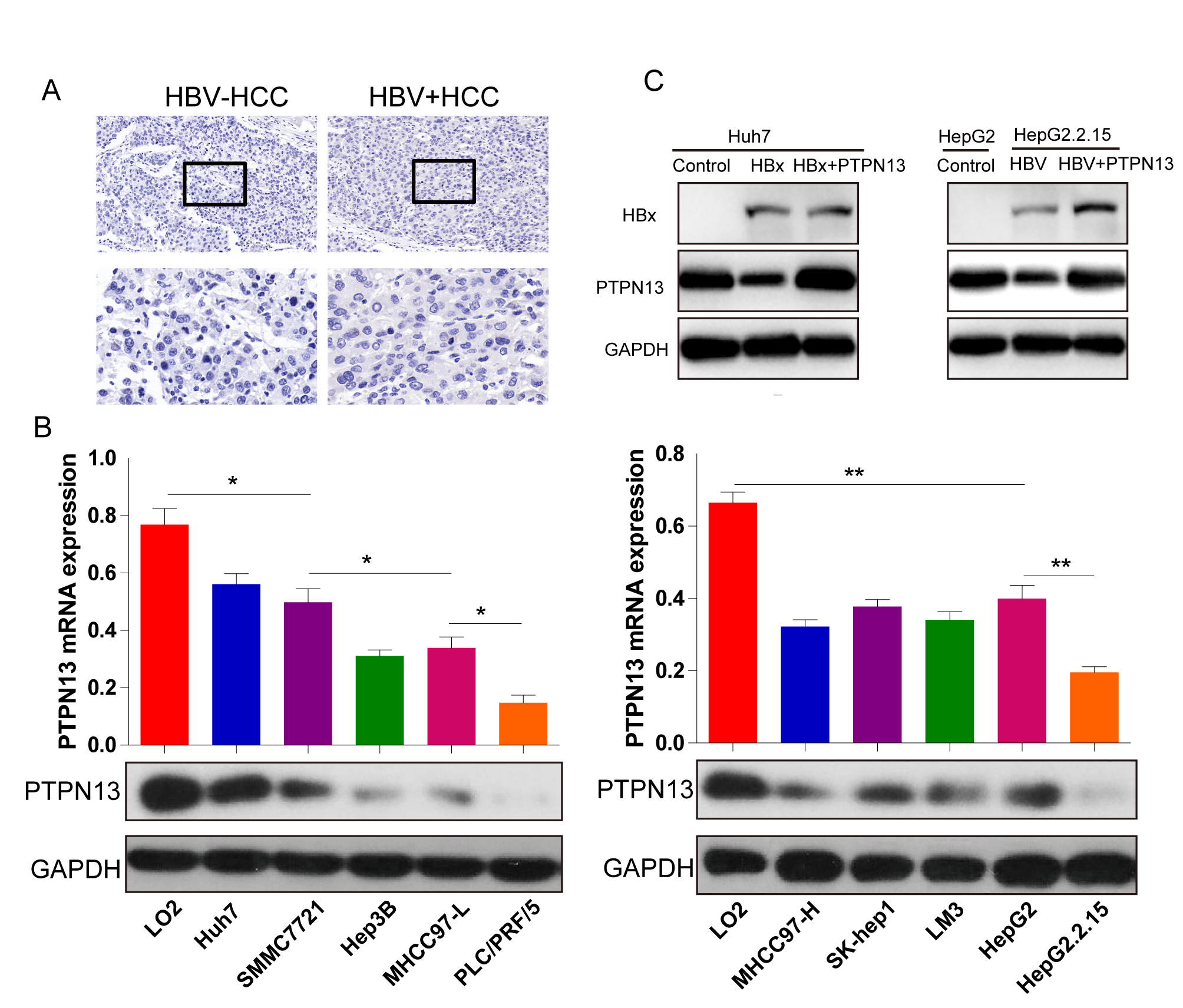

Supplement: Supplementary file 4 — Supplementary Figure 1 [file 41388_2020_1498_MOESM4_ESM.tif]

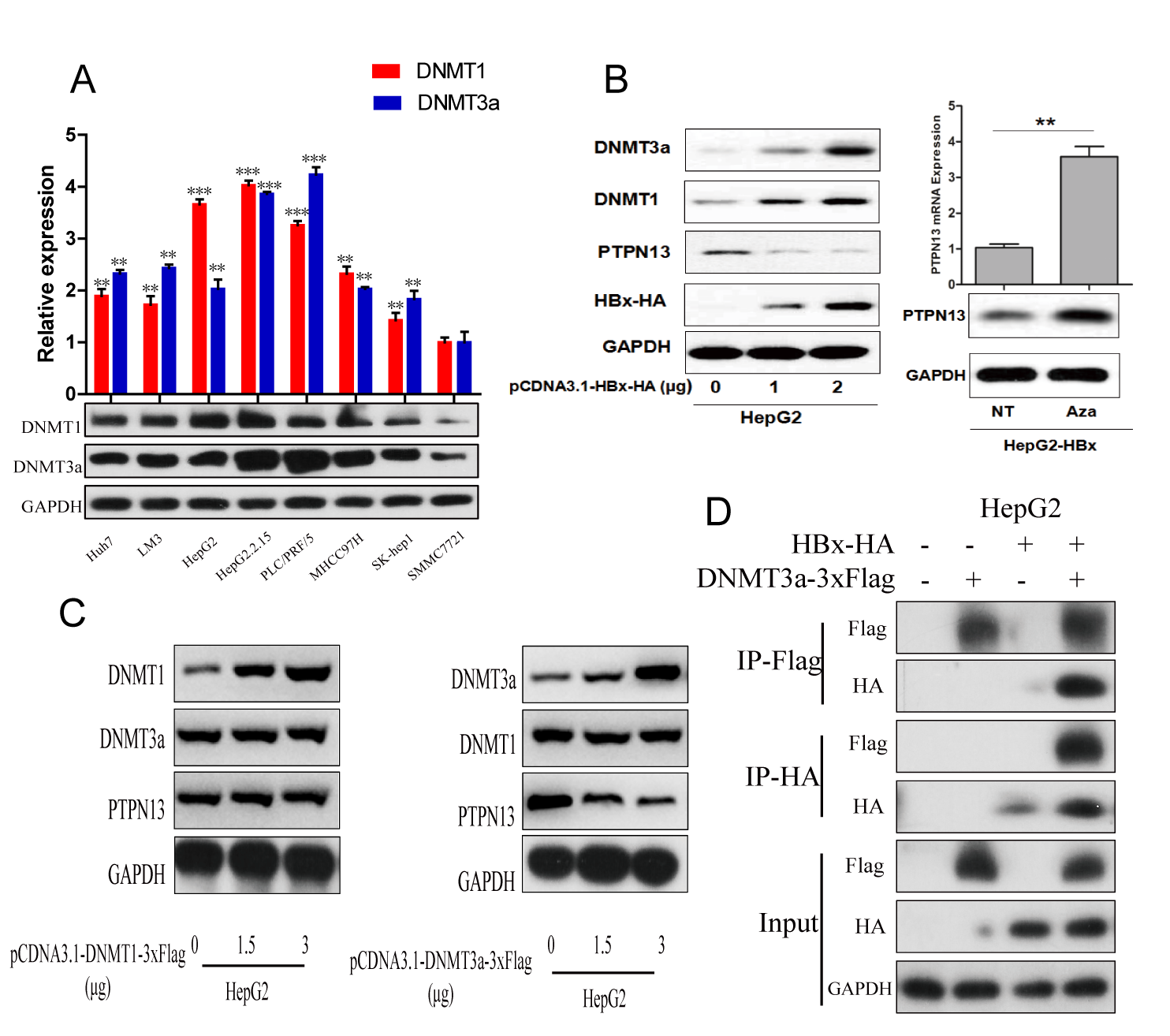

Supplement: Supplementary file 5 — Supplementary Figure 2 [file 41388_2020_1498_MOESM5_ESM.tif]

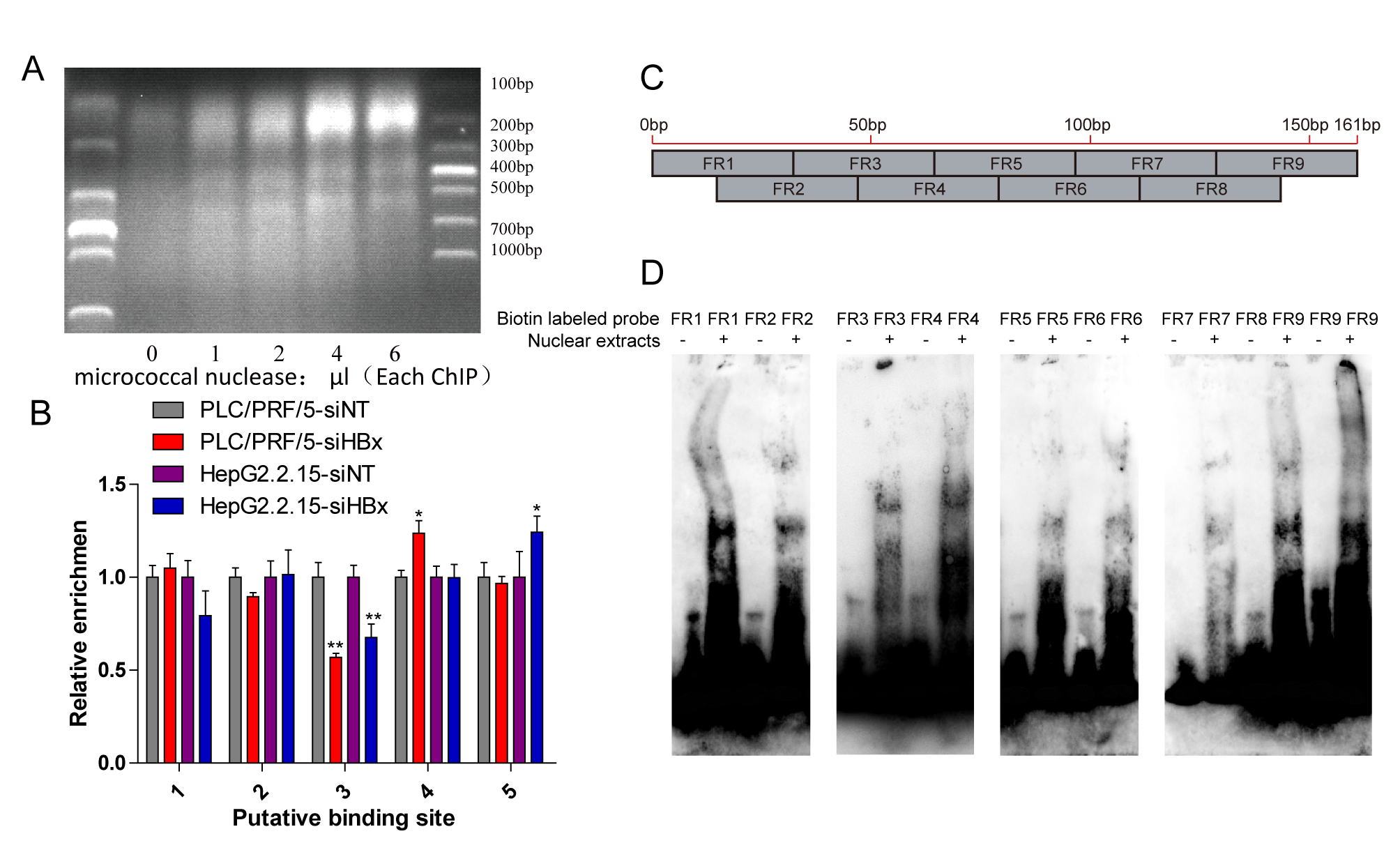

Supplement: Supplementary file 6 — Supplementary Figure 3 [file 41388_2020_1498_MOESM6_ESM.tif]

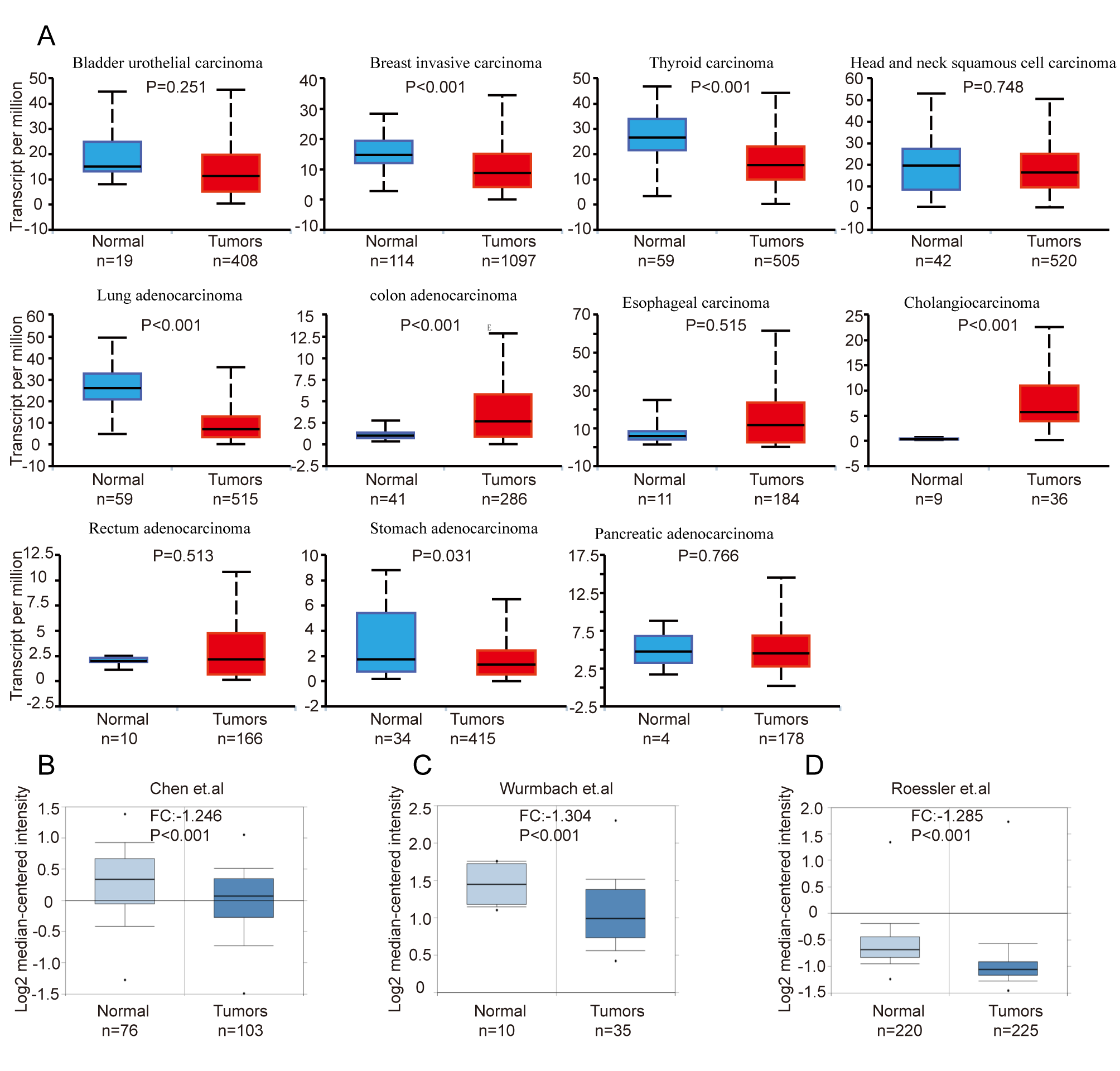

Supplement: Supplementary file 7 — Supplementary Figure 4 [file 41388_2020_1498_MOESM7_ESM.tif]

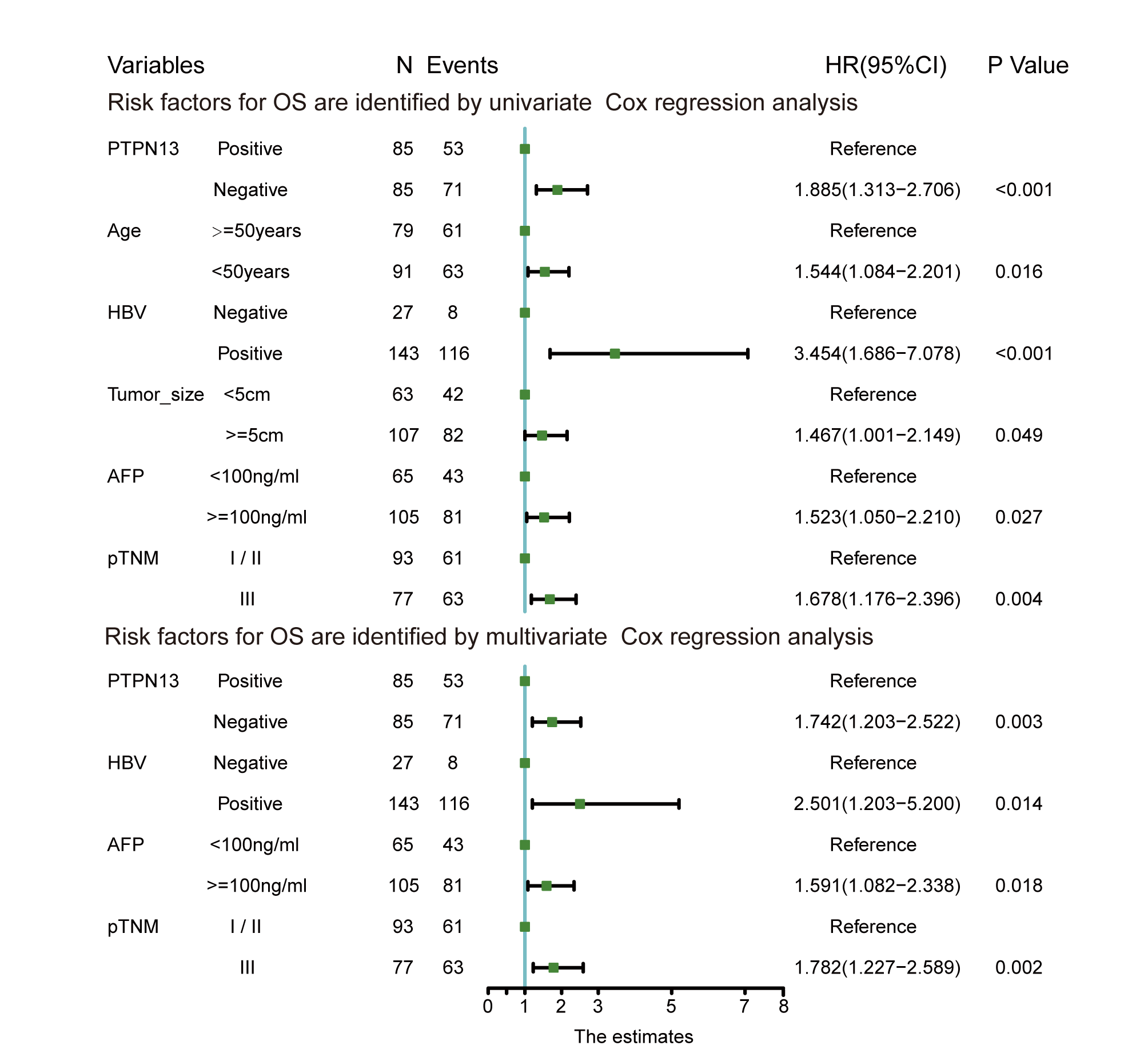

Supplement: Supplementary file 8 — Supplementary Figure 5 [file 41388_2020_1498_MOESM8_ESM.tif]

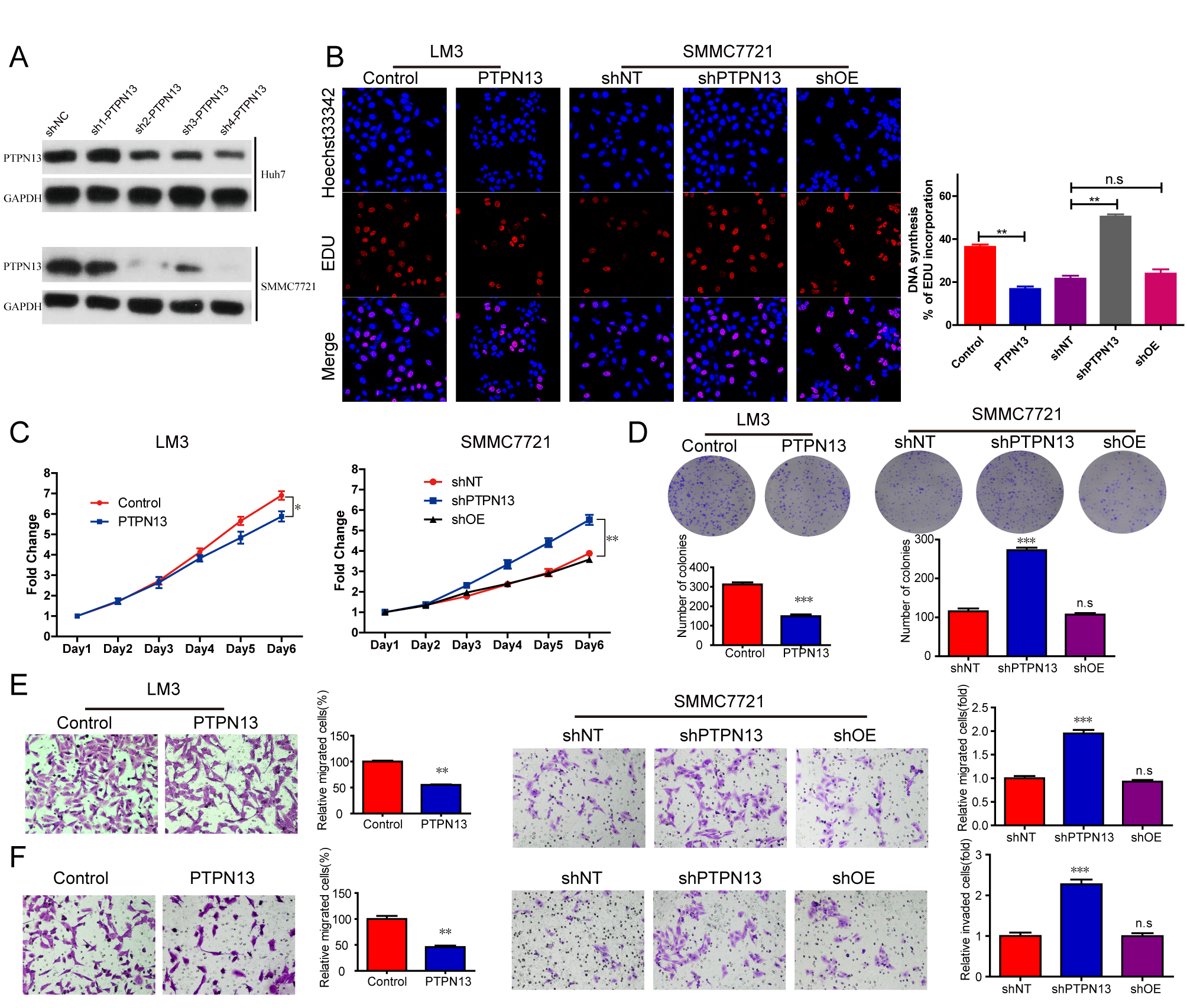

Supplement: Supplementary file 9 — Supplementary Figure 6 [file 41388_2020_1498_MOESM9_ESM.tif]

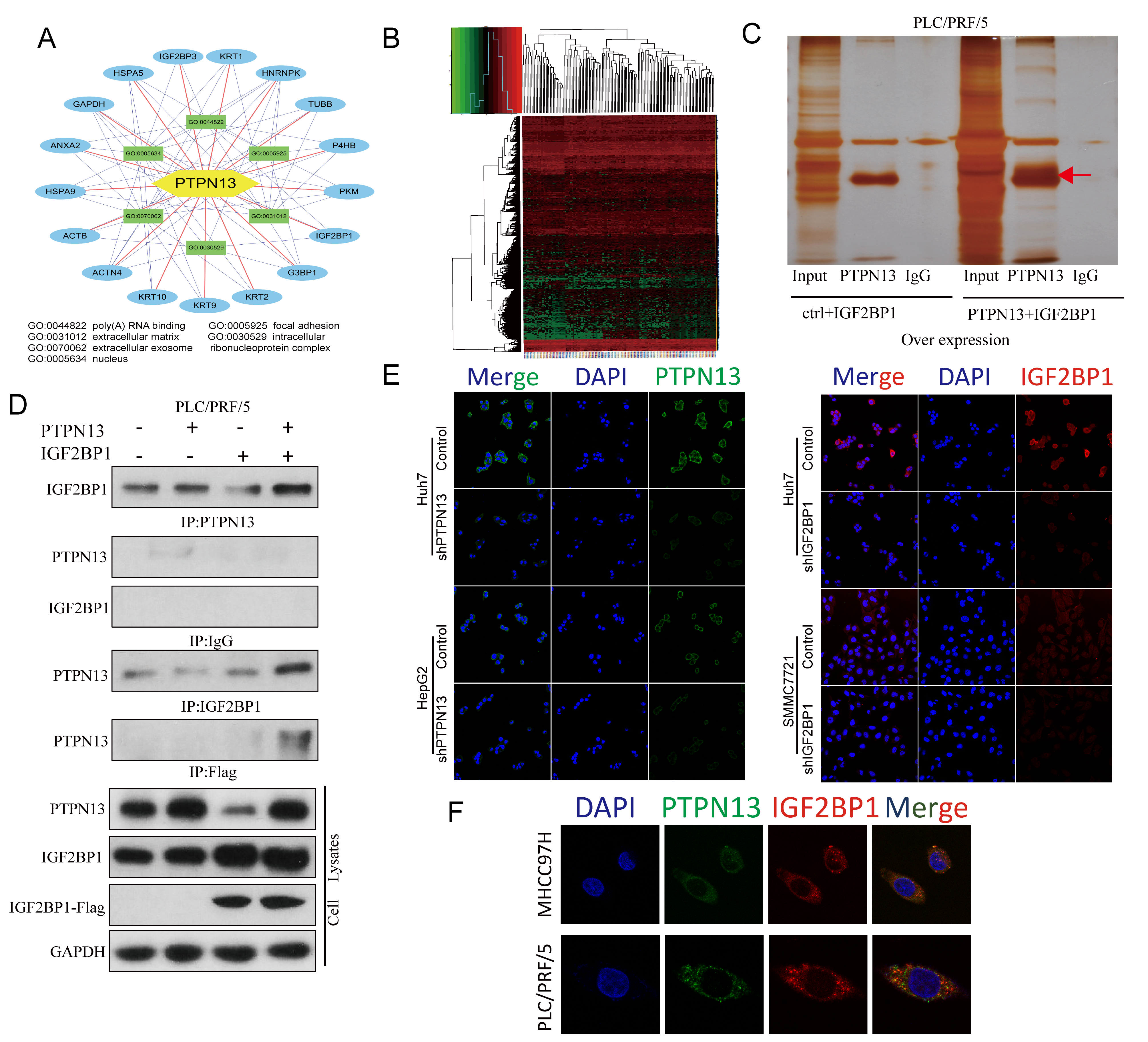

Supplement: Supplementary file 10 — Supplementary Figure 7 [file 41388_2020_1498_MOESM10_ESM.tif]

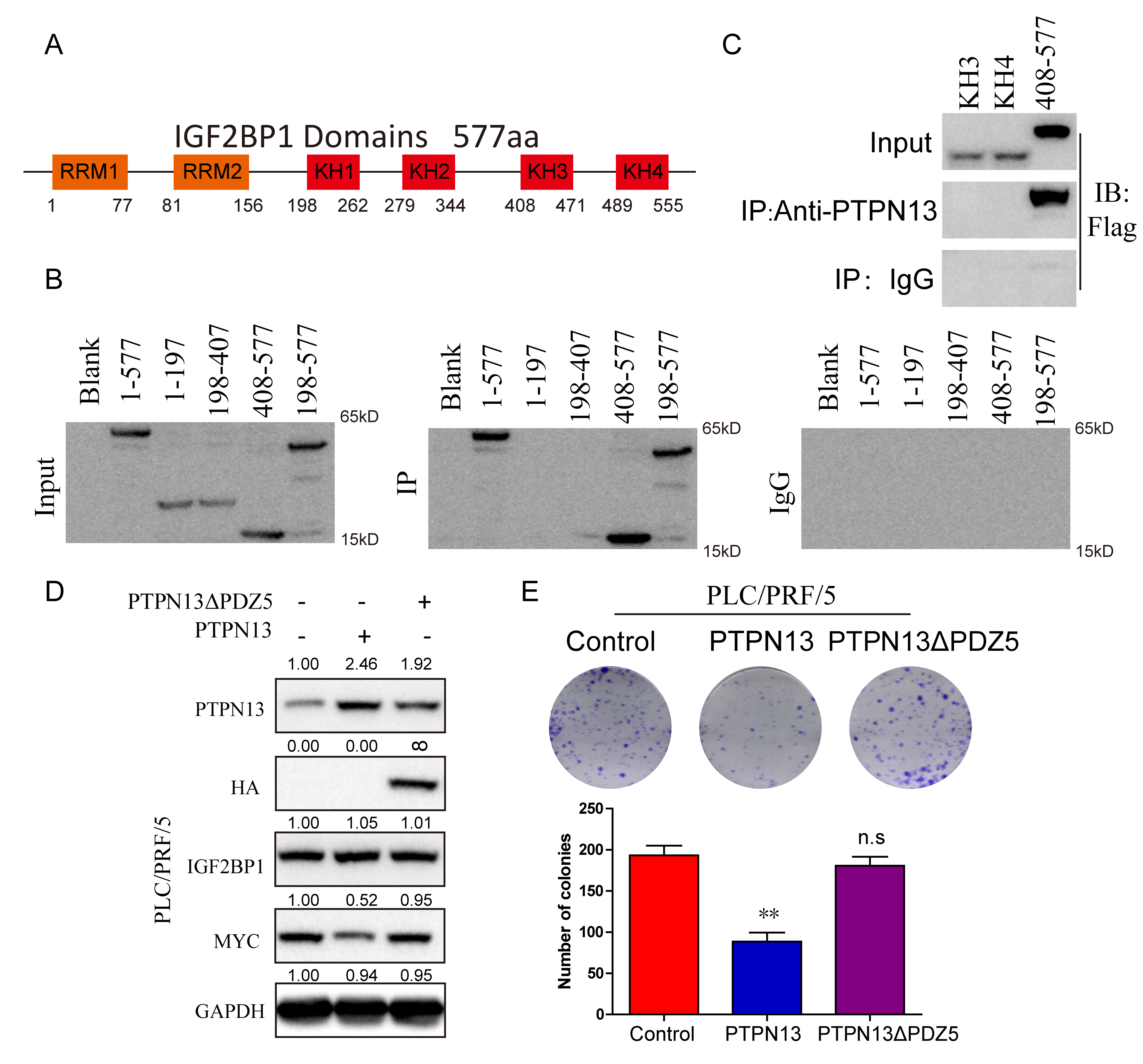

Supplement: Supplementary file 11 — Supplementary Figure 8 [file 41388_2020_1498_MOESM11_ESM.tif]

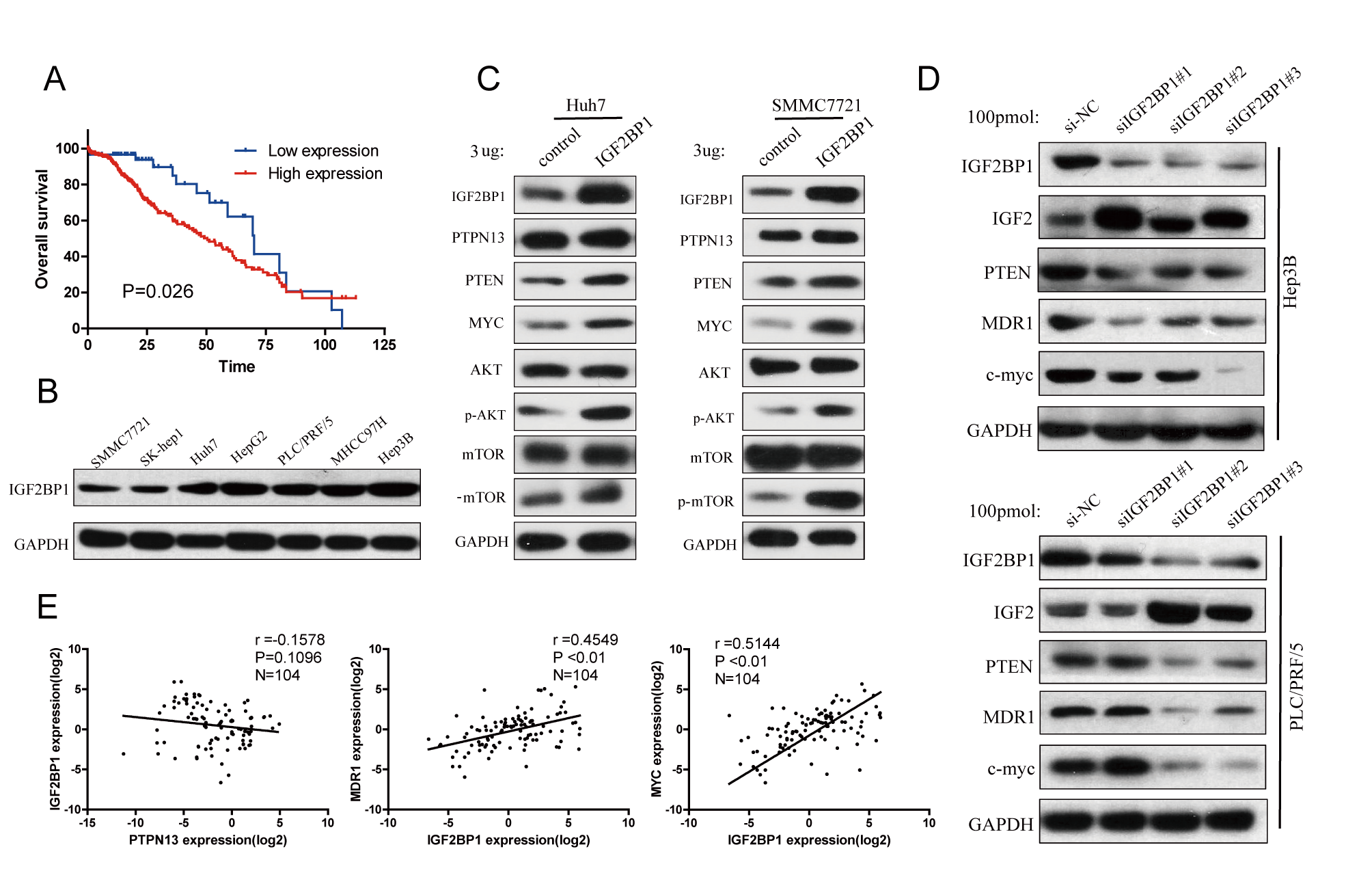

Supplement: Supplementary file 12 — Supplementary Figure 9 [file 41388_2020_1498_MOESM12_ESM.tif]

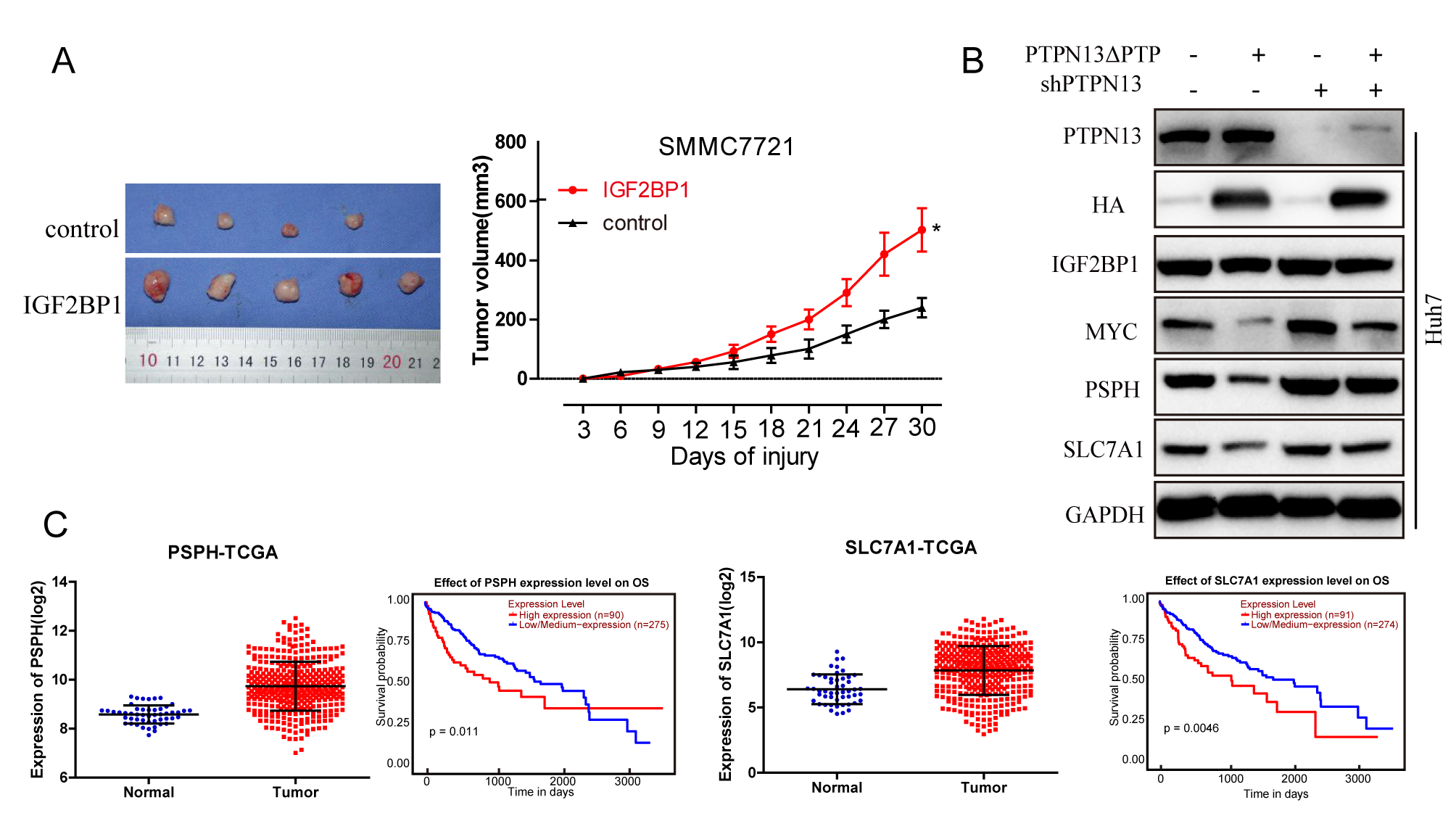

Supplement: Supplementary file 13 — Supplementary Figure 10 [file 41388_2020_1498_MOESM13_ESM.tif]

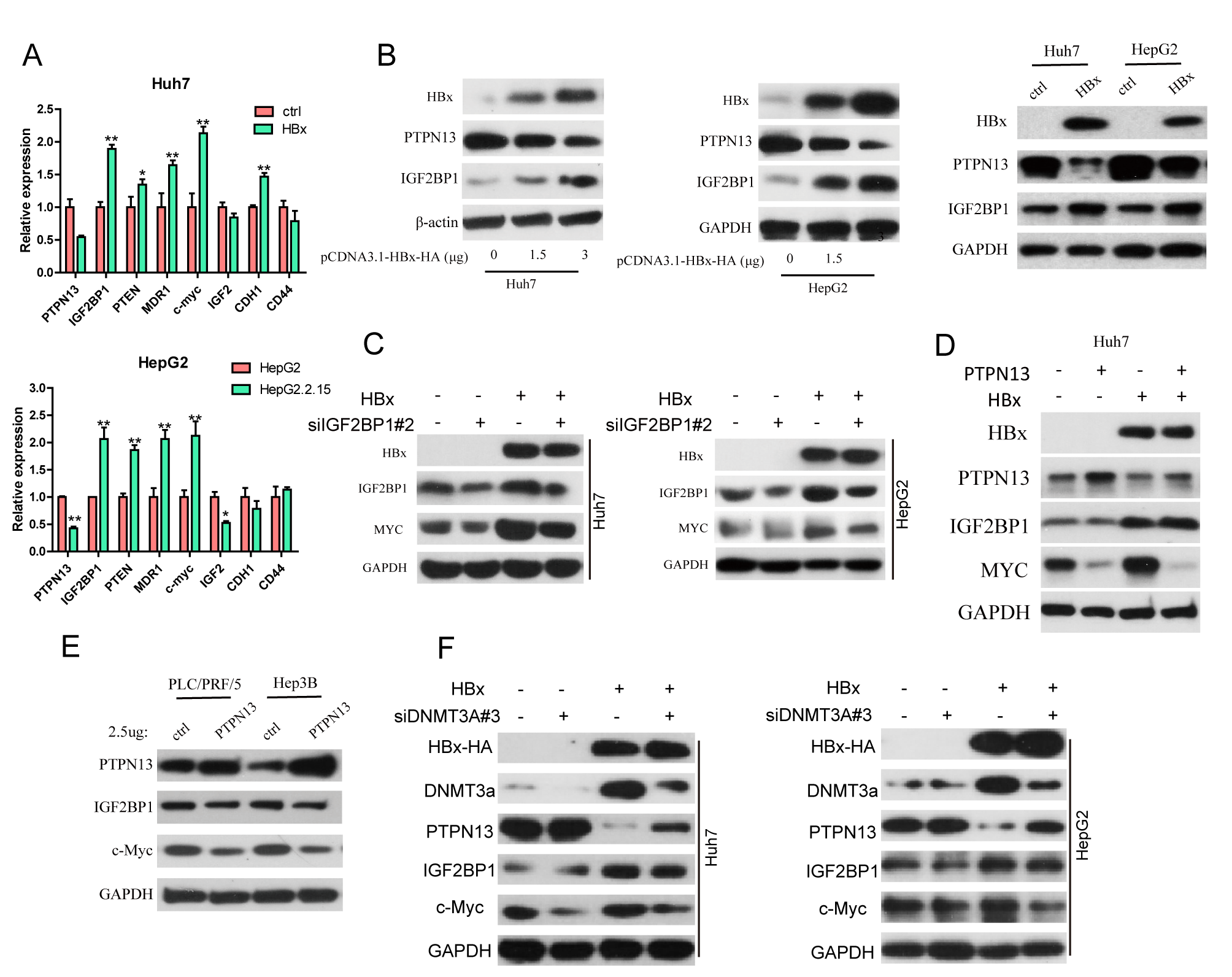

Supplement: Supplementary file 14 — Supplementary Figure 11 [file 41388_2020_1498_MOESM14_ESM.tif]
